# No Evidence that Selection on Synonymous Codon Usage Affects Patterns of Protein Evolution in Bacteria

**DOI:** 10.1093/gbe/evad232

**Published:** 2023-12-27

**Authors:** Ana Filipa Moutinho, Adam Eyre-Walker

**Affiliations:** School of Life Sciences, University of Sussex, Brighton, UK; School of Life Sciences, University of Sussex, Brighton, UK

**Keywords:** codon usage bias, natural selection, bacteria, protein evolution, mutation bias, biased gene conversion

## Abstract

Bias in synonymous codon usage has been reported across all kingdoms of life. Evidence suggests that codon usage bias is often driven by selective pressures, typically for translational efficiency. These selective pressures have been shown to depress the rate at which synonymous sites evolve. We hypothesize that selection on synonymous codon use could also slow the rate of protein evolution if a non-synonymous mutation changes the codon from being preferred to unpreferred. We test this hypothesis by looking at patterns of protein evolution using polymorphism and substitution data in two bacterial species, *Escherichia coli* and *Streptococcus pneumoniae*. We find no evidence that non-synonymous mutations that change a codon from being unpreferred to preferred are more common than the opposite. Overall, selection on codon bias seems to have little influence over non-synonymous polymorphism or substitution patterns.

**Significance**

Synonymous codon usage bias is a universal phenomenon often associated with selection to increase translational efficiency. Such selective pressures are thought to slow the rate at which synonymous sites evolve. Yet, no one has yet assessed whether it could also have an impact on the evolution of non-synonymous sites. Surprisingly, we find no evidence that selection on synonymous codon bias affects the pattern of non-synonymous polymorphism or substitution.

## Introduction

The genetic code is degenerate since 61 sense codons code for 20 amino acids, so most amino acids are encoded by more than one codon. These synonymous codons, however, are not equally used, a phenomenon known as codon usage bias (CUB) (Clarke 1970; Grantham, Gautier, Gouy, et al. 1980; Grantham, Gautier, Gouy and Mercier et al. 1980; Post and Nomura 1980). The origins of such biases have been extensively debated. Evidence suggests that mutation bias, biased gene conversion, and natural selection influence CUB. However, the extent to which each factor affects codon usage depends on the species. While mutation bias and biased gene conversion seem to dominate in some species (Muto and Osawa 1987; Ohama et al. 1990; Wright and Bibb 1992; Harrison and Charlesworth 2011), in others, such as *Escherichia coli*, selection plays a key role (Gouy and Gautier 1982; Sharp et al. 1993; Urrutia and Hurst 2001).

Various selective pressures have been proposed to affect synonymous sites, such as DNA stability, RNA stability, protein folding, gene regulation, and translational efficiency (Sharp and Li 1986; Eyre-walker and Bulmer 1993; Eyre-Walker 1996; Stoletzki and Eyre-Walker 2007; Plotkin and Kudla 2011). The last is the most widely studied, with extensive evidence supporting this hypothesis (Akashi 1995; Eyre-Walker 1996; Stoletzki and Eyre-Walker 2007). One of the earliest observations of selection for translation efficiency was the correlation between the use of certain synonymous codons and tRNA concentrations (Ikemura 1985; Bulmer 1987). According to this theory, selection favors the use of more frequent codons, the so-called major codons, as these may increase translation efficiency. Such selective pressure is expected to be stronger in highly expressed genes, a pattern observed in many species (Ikemura 1985; Sharp and Li 1986; Duret 2002). Moreover, evidence suggests that genes with high codon bias have lower rates of synonymous substitutions than genes with low codon bias (Sharp and Matassi 1994; Plotkin and Kudla 2011). These observations suggest that selection acts upon codon usage during translation, a pattern observed in multiple organisms, including bacteria (Sharp and Li 1987a, 1987b; Berg and Martelius 1995), yeast (Bennetzen and Hall 1982; Sharp and Li 1986) *Drosophila* (Shields et al. 1988; Sharp and Li 1989), and nematodes (Stenico et al. 1994).

The component of translation that selection is acting upon remains the subject of some debate (Akashi and Eyre-Walker 1998; dos Reis et al. 2004; Stoletzki and Eyre-Walker 2007). The use of certain codons could potentially affect the elongation rate, the cost of proofreading or the accuracy of translation (Bulmer 1991; Akashi and Eyre-Walker 1998; dos Reis et al. 2004; Stoletzki and Eyre-Walker 2007). However, as the initiation of translation is rate-limiting, selection on elongation is not to maximize the rate at which a particular gene is translated but to maximize the overall efficiency of translation by using ribosomes most efficiently. Hence, using optimal codons does not increase the expression of a gene, but it can increase the growth rate (Bulmer 1991; Weissman et al. 2021). Evidence of selection for translation accuracy is also widespread. In a ground-breaking study, Akashi (1994) showed that the protein's most important amino acid sites had a higher bias in *Drosophila melanogaster*, an observation also made in *E. coli* (Stoletzki and Eyre-Walker 2007). The observed correlation between CUB and gene length in *E. coli* (Eyre-Walker 1996) is also consistent with accuracy—it is more energetically expensive to make mistakes translating longer genes.

While the selective determinants of CUB remain debated, one thing is clear: Selection on codon usage depresses the rate of synonymous substitution in many species (Sharp and Li 1987a, 1987b; Sharp and Li 1989; Stenico et al. 1994; Berg and Martelius 1995). At the level of coding sequence evolution, however, little is known about the impact of CUB. We hypothesize that selection for codon usage could also impede the rate of non-synonymous substitutions if two amino acids vary substantially in their codon usage. Such an effect is expected if selection on codon usage is not too weak compared to the cost of the amino acid change. In this scenario, a non-synonymous mutation will be favored by selection if it changes from a sub-optimal to an optimal codon, whereas the opposite will be disfavored. For example, consider a non-synonymous substitution between Lysine (Lys) and Glutamine (Gln) in *E. coli*. As amino acids tend to use codons with high codon usage more frequently, we expect that a non-synonymous substitution changing Lys to Gln will generally involve a mutation from AAA, the preferred codon for Lys, to CAA, the unpreferred codon for Gln. One would therefore expect that selection on synonymous codon use will disfavor this mutation, thus reducing the levels of non-synonymous polymorphism and substitution. We investigate whether selection on synonymous codon use affects patterns of non-synonymous polymorphism and substitution in two bacterial species: *E. coli*, because there is extensive evidence for selection on codon usage (Hershberg and Petrov 2008), and *Streptococcus pneumoniae*, as it is inferred to have one of the strongest selective pressures on codon usage among bacteria (Sharp et al. 2005).

## Results

### Polymorphisms

We devised a series of tests to investigate whether selection on synonymous codon usage affects protein evolution. First, we assessed the impact of CUB on segregating non-synonymous polymorphisms by considering all amino acid pairs separated by a single non-synonymous mutation. For example, let us consider sites that are polymorphic for Glutamine (Gln) and Lysine (Lys), using a closely related species to infer the ancestral state. Thus, each amino acid pair was composed of the ancestral and the derived allele. We consider the mutations from Gln → Lys and Lys → Gln separately to account for the differences in selective pressures between amino acids, mutation bias and biased gene conversion. As a measure of codon usage, we use the relative synonymous codon usage (RSCU, i.e. the observed frequency of each codon divided by the frequency expected when assuming equal usage for all synonymous codons within an amino acid (Sharp et al. 1986)) of codons in highly expressed genes, for which codon bias and selection are thought to be strongest. We consider a change from an unpreferred (low RSCU) to a preferred (high RSCU) codon when the RSCU value increases, and a change from a preferred to unpreferred codon when the RSCU value decreases. Unfortunately, RSCU values for codons of different degeneracy are not comparable. While the expected value of RSCU is one when there is no selection (or mutation bias and biased gene conversion), it has different maximum values when there is selection—two for a 2-fold and four for a 4-fold. We therefore divide our analyses into comparisons between amino acids where both amino acids are 2-fold degenerate and comparisons in which both amino acids are 4-fold degenerate.

We predict that non-synonymous polymorphisms changing toward more preferred codons should on average be more favored by selection. So, in the case of Gln → Lys in *E. coli*, as AAA and AAG are, respectively, the preferred and unpreferred codons for Lys, and CAG and CAA are, respectively, the preferred and unpreferred codons for Gln, we expect the numbers of CAA → AAA polymorphisms, per CAA site, should be greater than CAG → AAG, per CAG site. We can quantify the bias in favor of CAA → AAA in terms of an odds ratio, where we consider polymorphic sites inferred to be CAA → AAA ($N_{\mathrm{CAA}\to \mathrm{AAA}}$), CAG → AAG ($N_{\mathrm{CAG}\to \mathrm{AAG}}$), and monomorphic sites that are CAA ($N_{\mathrm{CAA}})$ and CAG ($N_{\mathrm{CAG}})$ (see supplementary File S1, Supplementary Material online):


(1) $$Y_{Gln\to Lys}=\frac{N_{\mathrm{CAA}\to \mathrm{AAA}}N_{\mathrm{CAG}}}{N_{\mathrm{CAG}\to \mathrm{AAG}}N_{\mathrm{CAA}}}.$$

If both amino acids have more than two synonymous codons, we contrast the number of polymorphisms between the codons which lead to the biggest increase in RSCU ($\mathrm{RSC}U_{\max}$), to those which lead to the biggest decrease ($\mathrm{RSC}U_{\min}$). For example, when considering Pro → Ala in *E. coli*, CCC → ACC changes the RSCU in highly expressed genes from 0.05 to 1.75, and we contrast this to CCG → ACG which changes the RSCU from 2.85 to 0.17. In this case,


(2) $$Y_{Pro\to Ala}=\frac{N_{\mathrm{CCC}\to \mathrm{ACC}}N_{\mathrm{CCG}}}{N_{\mathrm{CCG}\to \mathrm{ACG}}N_{\mathrm{CCC}}}.$$

We combine *Y* estimates across genes using the Cochran–Mantel–Haenszel method (Mantel 1963) to avoid Simpson's paradox; this yields a joint estimate of the OR. As *Y* is a ratio of ratios, we use the logarithm scale and to differentiate it from a related statistic, which we introduce below, that uses divergence between species data, we refer to the statistic as *Y*_pol_.

**Fig. 1.**
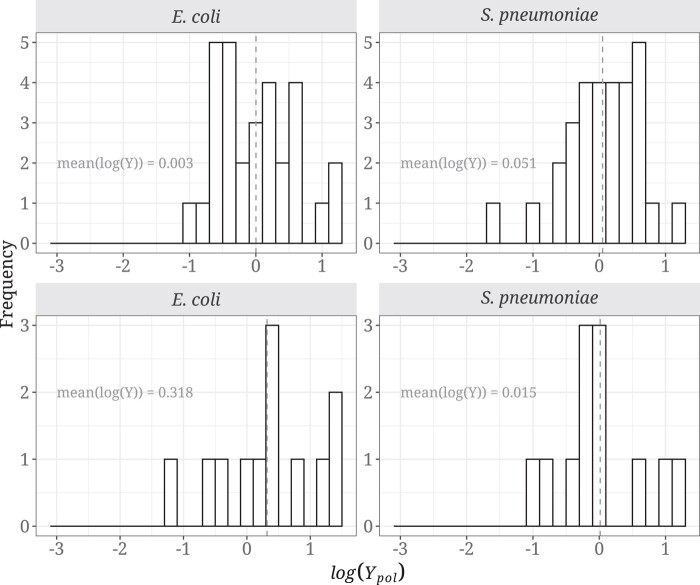
Distribution of log(*Y*_pol_) in *E. coli* and *S. pneumoniae* for 2-fold (top) and 4-fold (bottom) amino acid combinations. The dashed line represents the mean value of the distribution. The binning size was set at 0.2. This analysis was performed with 30 and 12 amino acid mutations for 2-fold and 4-fold pairs, respectively, in each species.

Positive log(*Y*_pol_) values mean that there are more non-synonymous polymorphisms changing the codon from an unpreferred (low RSCU) to a preferred state (high RSCU), than from a preferred to an unpreferred state. We hypothesize that if selection on synonymous codon use is affecting patterns of protein evolution, log(*Y*_pol_) should on average be greater than zero. To polarize the polymorphisms, we used two different outgroups to check for differences between them: *Escherichia albertii* and *Escherichia fergusonii* for *Escherichia coli*, and *Streptococcus pseudopneumoniae* and *Streptococcus mitis* for *Streptococcus pneumoniae*. We find no support for our prediction, as the distribution of log(*Y*_pol_) values is not significantly different from 0 in either species (Fig. 1), for either 2-fold (*E. coli*: mean = 0.003, *P* = 0.981; *S. pneumoniae*: mean = 0.051, *P* = 0.641) or 4-fold degenerate amino acids (*E. coli*: mean = 0.318, *P* = 0.199; *S. pneumoniae*: mean = 0.015, *P* = 0.937) when using as outgroups *E. albertii* and *S. pseudopneumoniae* for *E. coli* and *S. pneumoniae*, respectively. Similar results were obtained when using *E. fergusonii* and *S. mitis* as outgroups (supplementary fig. S1, Supplementary Material online), we therefore performed the rest of the polymorphism analyses with the closest outgroup of each species (*E. albertii* and *S. pseudopneumoniae* for *E. coli* and *S. pneumoniae*, respectively: *d_S_*_[*E. coli*_  _−_  *_E. albertii_*_]_: 0.262, *d_S_*_[*E. coli*_  _−_  *_E. fergusonii_*_]_: 0.270, *d_S_*_[*S. pneumoniae*_  _−_  *_S. pseudopsneumoniae_*_]_: 0.146, *d_S_*_[*S. pneumoniae*_  _−_  *_S. mitis_*_]_: 0.216; where *d_S_* represents the synonymous genetic divergence between the each species comparison).

We also predict that log(*Y*_pol_) should be correlated to the difference in the RSCU values between the ancestral and the derived allele. For example, consider the case of Gln → Lys, involving CAA → AAA and CAG → AAG codon mutations, and Glu → Lys, involving GAA → AAA and GAG → AAG codon mutations. In *E. coli*, AAA is used 73% of the time in highly expressed genes, AAG 27%, CAA 19%, CAG 81%, GAA 75%, and GAG 25%. This means that a mutation from Gln → Lys involves a large change in RSCU whereas Glu → Lys does not. Hence, we might expect Gln → Lys to be more affected by selection on synonymous codon use, than Glu → Lys and *Y*_pol_ for Gln → Lys to be greater than for Glu → Lys. We measure the difference in RSCU values as:


(3) $$\Delta \mathrm{RSCU}=\frac{RSCU_{derived(max)}/RSCU_{ancestral(max)}}{RSCU_{derived(min)}/RSCU_{ancestral(min)}}$$

where $\mathrm{RSC}U_{\mathrm{derived}(\max)}\;$ and $\mathrm{RSC}U_{\mathrm{ancestral}(\max)}$ represent the RSCU values of the derived and ancestral allele, respectively, of the codon pair leading to the largest change in RSCU (max); and $\mathrm{RSC}U_{\mathrm{derived}(\min)}\;$ and $\mathrm{RSC}U_{\mathrm{ancestral}(\min)}$ represent the RSCU values of the derived and ancestral allele, respectively, of the codon pair leading to the smallest change in RSCU (min). For Gln → Lys, this is:


(4) $$\Delta \mathrm{RSCU}=\frac{RSCU_{AAA}/RSCU_{CAA}}{RSCU_{AAG}/RSCU_{CAG}}.$$

**Fig. 2.**
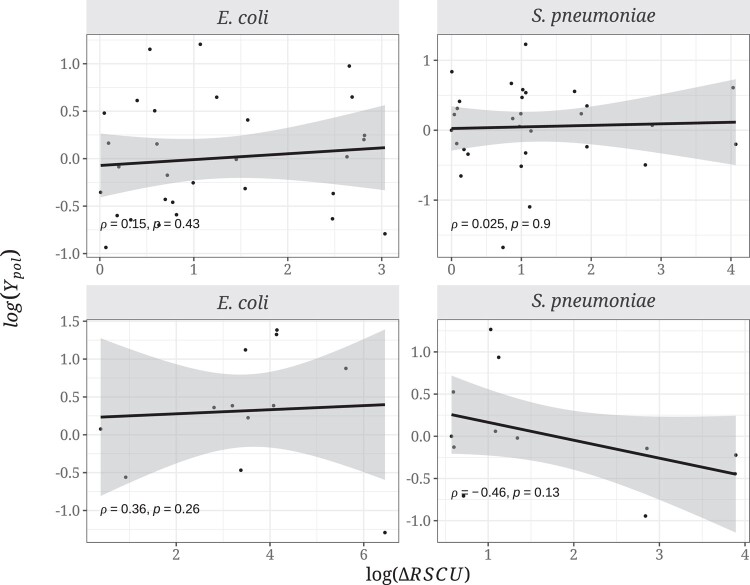
Relationship between the ratio of the differences in RSCU values between codons (log(ΔRSCU)) and log(*Y*_pol_) in *E. coli* and *S. pneumoniae* for 2-fold (top) and 4-fold (bottom) degenerate amino acid combinations. A linear model was fitted to the data and is represented with the dark line along with the Spearman's correlation coefficient and the respective significance values. Legend as in Fig. 1.

Contrary to our expectations, we observed no significant correlations between log(*Y*_pol_) and the log(ΔRSCU) (Fig. 2) for either 2-fold (*E. coli*: Spearman's *ρ* = 0.15, *P* = 0.43; *S. pneumoniae*: *ρ* = 0.025, *P* = 0.90) or 4-fold codons (*E. coli*: *ρ* = 0.36, *P* = 0.26; *S. pneumoniae*: *ρ* = *−*0.46, *P* = 0.13).

### Substitutions

We can perform a similar analysis for substitutions between species. As in the analysis at the polymorphism level, we predict that non-synonymous substitutions changing toward preferred codons should on average be more favored by selection. To test this hypothesis, we focus our analysis on the sites that are fixed in the populations of *E. coli* and *S. pneumoniae*. Codon substitutions were then polarized according to the two outgroups for each species: *E. albertii* and *E. fergusonii* for *E. coli*, and *S. pseudopneumoniae* and *S. mitis* for *S. pneumoniae*, keeping only the sites where the two outgroups had the same allele. Thus, using again the example of Gln → Lys in *E. coli*, we only consider a codon substitution from CAA to AAA if both *E. albertii* and *E. fergusonii* are CAA and *E. coli* is AAA. By applying the same statistics described above (see equations (1) and (2)) to the case of Gln → Lys, we can quantify the bias in favor of CAA → AAA with an odds ratio, where we consider codon substitutions inferred to be CAA → AAA ($N_{CAA\to AAA}$), CAG → AAG ($N_{CAG\to AAG}$), and fixed sites among the tree species (i.e. the three species have the same allele) that are CAA ($N_{CAA})$ and CAG ($N_{CAG})$ (see supplementary File S2, Supplementary Material online). We call this statistic using divergence between species $Y_{\mathrm{div}}$.

**Fig. 3.**
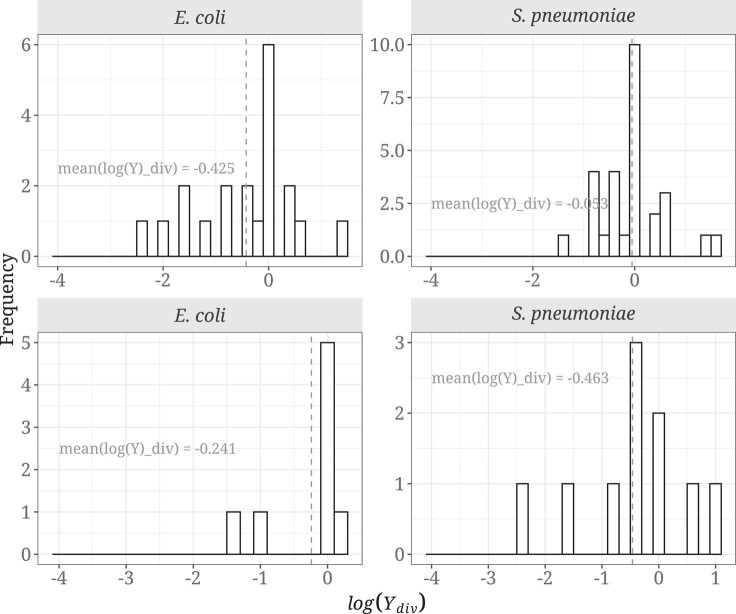
Distribution of $\log(Y_{\mathrm{div}})$ in *E. coli* and *S. pneumoniae* for 2-fold (top) and 4-fold (bottom) amino acid combinations. This analysis was performed with 20 and 8 amino acid mutations for 2-fold and 4-fold pairs, respectively, in *E. coli*, and 28 and 10 amino acid mutations for 2-fold and 4-fold pairs, respectively, in *S. pneumoniae*; $\log(Y_{\mathrm{div}})$ was undefined for some amino acid combinations. Legend as in Fig. 1.

We predict log(*Y*_div_) to be greater than zero if selection on synonymous codon use is affecting patterns of non-synonymous substitutions. However, we find no support for this prediction, instead observing the opposite pattern as the distribution of $\log(Y_{\mathrm{div}})$ is slightly skewed toward negative values (Fig. 3), for both 2-fold (*E. coli*: mean = −0.425, *P* = 0.057; *S. pneumoniae*: mean = −0.241, *P* = 0.270) and 4-fold degenerate amino acids (*E. coli*: mean = −0.053, *P* = 0.677; *S. pneumoniae*: mean = −0.463, *P* = 0.168).

**Fig. 4.**
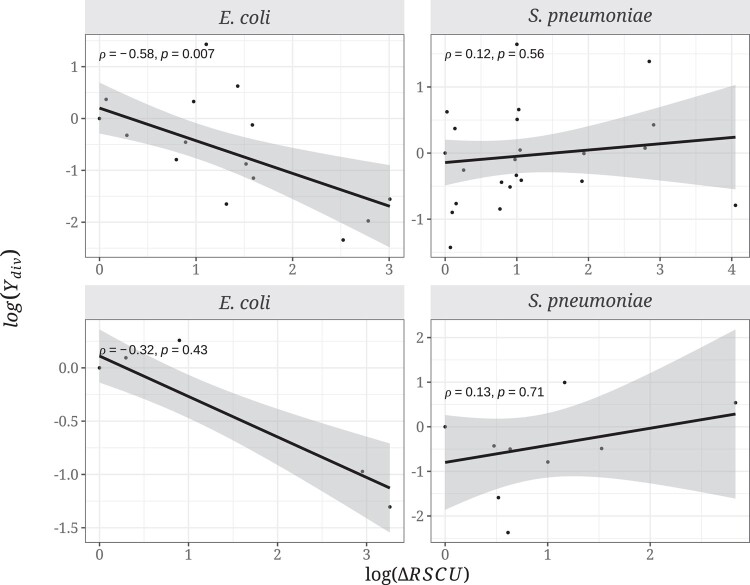
Relationship between the ratio of the differences in RSCU values between codons (log(ΔRSCU)) and $\log(Y_{\mathrm{div}})$ in *E. coli* and *S. pneumoniae* for 2-fold (top) and 4-fold (bottom) degenerate amino acid combinations. Legend as in Fig. 2.

Similarly, we also predict that $\log(Y_{\mathrm{div}})$ should be positively correlated to the strength of selection acting on codon usage at the divergence level. We used the same approach as above for the polymorphism data, using the same RSCU values as in the polymorphism analysis for the focal species and the RSCU values estimated using the same set of highly expressed genes in the closest outgroup (*E. albertii* and *S. pseudopneumoniae* for *E. coli* and *S. pneumoniae*, respectively). Contrary to our expectations, we found no significant correlation between $\log(Y_{\mathrm{div}})$ and the log(ΔRSCU) in *S. pneumoniae* (Fig. 4; 2-fold: Spearman's *ρ* = 0.12, *P* = 0.56; 4-fold: *ρ* = 0.13, *P* = 0.71), while in *E. coli*, we observed the opposite pattern, with $\log(Y_{\mathrm{div}})$ being negatively correlated with log(ΔRSCU) (Fig. 4; 2-fold: Spearman's *ρ* = −0.58, *P* = 0.007; 4-fold: *ρ* = −0.32, *P* = 0.43).

### Gene Expression

It is possible that we have failed to detect a strong effect of synonymous codon selection on protein evolution because many genes are subject to weak selection on codon usage, and these add noise to our analysis. To investigate this, we divided our genes into equal sized groups according to expression level, since selection on codon bias is known to be stronger in more highly expressed genes (Post and Nomura 1980; Gouy and Gautier 1982; Sharp et al. 2010). As we do not have gene expression data for all genes in our data set, we used the level of codon bias of a gene, as quantified by the codon adaptation index (CAI) (Sharp and Li 1987a, 1987b), as a proxy for gene expression. These two measures are significantly positively correlated for those genes for which we have data (supplementary fig. S2, Supplementary Material online; *E. coli*: Spearman's *ρ* = 0.52, *P* < 2.2e^−16^; *S. pneumoniae*: *ρ* = 0.23, *P* < 2.2e^−16^).

**Fig. 5.**
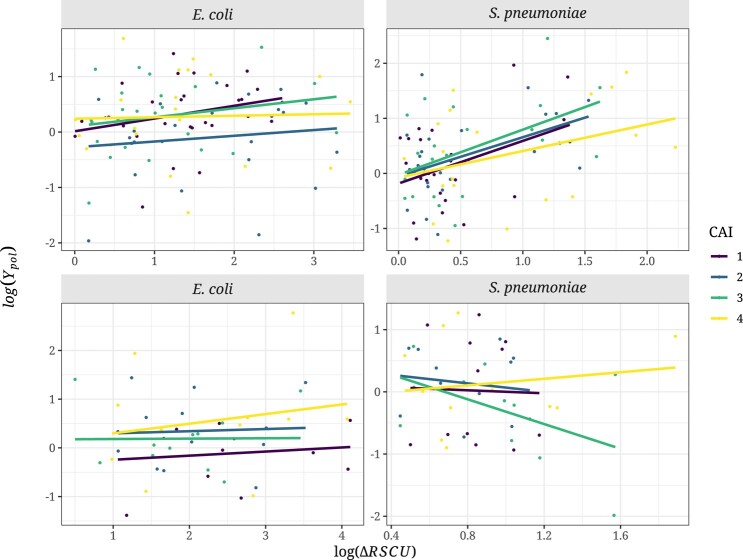
Relationship between log(*Y*_pol_) and log(ΔRSCU) and for *E. coli* and *S. pneumoniae* in each gene expression category for 2-folds (top) and 4-folds (bottom). A linear model was fitted to the data and each relationship is colored according to the respective CAI category (category 1: low CAI value; category 4: high CAI value). The number of polymorphisms used for this analysis can be found in supplementary table S4, Supplementary Material online.

**Fig. 6.**
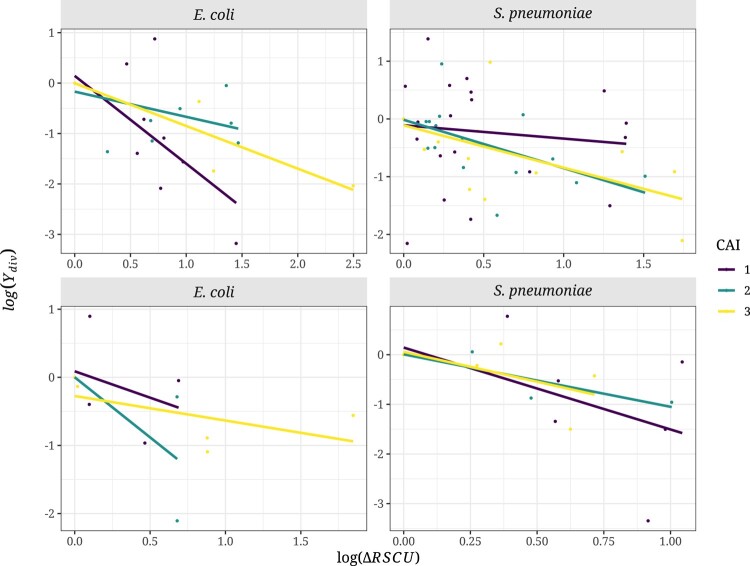
Relationship between $\log(Y_{\mathrm{div}})$ and the ratio of the differences in RSCU values between codons (log(ΔRSCU)) in *E. coli* and *S. pneumoniae* in each CAI category for 2-fold (top) and 4-fold (bottom) degenerate amino acid combinations. Legend as in Fig. 5. The number of substitutions used for this analysis can be found in supplementary table S5, Supplementary Material online.

We expect the slope of the relationship between log(*Y*_pol_), $\log(Y_{\mathrm{div}})$ and log(ΔRSCU) to be greater for more highly expressed and high CAI genes, because highly expressed genes are subject to stronger selection on codon bias. However, we find no evidence for this (Figs. 5 and 6). By running a linear model, we find no interaction between the slope of the relationship and the CAI category for either species, degeneracy of codon, for the polymorphism or divergence data (supplementary table S3, Supplementary Material online). Surprisingly, the relationship between log(*Y*_div_) and log(ΔRSCU) is significantly negative for 2-fold codons in both species, the opposite of what we expect.

## Discussion

We predict that selection on synonymous codon use should affect the level of non-synonymous polymorphism and substitution. However, we find no evidence of this either at the polymorphism or divergence levels in two bacterial species, *E. coli* and *S. pneumoniae*, both of which are believed to be subject to selection on CUB (Sharp et al. 2005). We do not find that non-synonymous variants changing from an unpreferred to a preferred codon are more prevalent than the opposite at either the polymorphism (Fig. 1) or divergence levels (Fig. 3), and there is no correlation with the strength of CUB (Figs. 2 and 4). Furthermore, we find no effect of gene expression in the analysis at the polymorphism and substitution levels (Figs. 5 and 6; supplementary table S3, Supplementary Material online).

It is perhaps surprising that selection on codon usage does not affect protein evolution, given that it seems to have substantial impact on the rate of synonymous substitution, as shown in studies with much less data (Sharp and Li 1987a, 1987b; Berg and Martelius 1995; Smith and Eyre-Walker 2001). There might be several explanations for this. First, it is possible that we do not have the power to detect an effect. To assess the power of our analysis, we considered the conditions under which log(*Y*_pol_) would be significantly greater than zero. We focus on this statistic given that it is the most fundamental in our analyses and probably has the greatest power. We find that if the expected value of *Y*_pol_ was 1.4—i.e. selection on synonymous codon usage increased the number of non-synonymous mutations that increased the RSCU value by 40% relative to the opposite direction—then we would have a 95% chance of detecting the effect at the 5% level for 2-folds in both *E. coli* using *E. fergusonii* as an outgroup and *S. pneumoniae* using *S. mitis* as the outgroup. We would therefore seem to have power to detect a moderate effect. However, when we estimate the strength of selection that is likely to be acting upon synonymous codon usage in both species, we find that it is only likely to increase the number of polymorphisms by about 10% for those increasing RSCU relative to those decreasing the RSCU in *E. coli* and by only 4% in *S. pneumoniae*; i.e. the expected value of log(*Y*_pol_) is 0.10 and 0.40 for *E. coli* and *S. pneumoniae*, respectively. Under these conditions, we only have a 30% chance of detecting a significant effect in *E. coli* and a 14% chance in *S. pneumoniae*. So, although selection appears to act upon synonymous codon usage in both species, it is not strong enough, and different enough between the codons of amino acids to have much influence. Our other analyses probably have less power since we are dividing the data between categories, and we have relatively few substitutions between species.

There are, however, some alternative explanations for why we do not observe a significant effect of selection on codon usage upon patterns of amino acid evolution which are worth mentioning. First, we have assumed that there is directional selection for preferred codons, and that the system is in a mutation–selection–drift equilibrium—selection favors certain codons but mutation and drift maintain some proportion of unpreferred codons. This is the model that has dominated this field since the pioneering work of Li (1987) and Bulmer (1991). There are two lines of evidence in favor of this model. First, codon bias is stronger in highly expressed genes. Second, rates of synonymous substitution are lower in highly expressed genes (Sharp and Li 1987a, 1987b; Smith and Eyre-Walker 2001), although, it has been suggested that this might be due to lower mutation rates in highly expressed genes (Berg and Martelius 1995; Eyre-Walker and Bulmer 1995). Third, the site frequency spectrum seems to be consistent with a mutation–selection–drift model (Smith and Eyre-Walker 2001). However, there are two alternative theories for why unpreferred codons exist. The first theory states that some unpreferred codons are used because the same sequence can have different functions—there is antagonistic pleiotropy (Akashi and Eyre-Walker 1998). Such a phenomenon has been well-characterized in *E. coli*, where the weaker bias in codon usage at the start of the genes has been attributed to selection against secondary structure in the mRNA (Eyre-walker and Bulmer 1993; Kudla et al. 2009). If antagonistic pleiotropy is prevalent, a low RSCU codon might be fitter at a particular site than a high RSCU codon. So, although high RSCU codons tend to be fitter than low RSCU codons, considerable deviations from this norm might obscure the patterns we predict. Second, codon usage might be subject to stabilizing rather than directional selection. Indeed, experiments with yeast have demonstrated that the average time for the ribosome to move through a codon is not correlated to the RSCU value or the concentration of the tRNA matching the codon (Qian et al. 2012). If selection is stabilizing, changing a preferred to unpreferred codon is equally strongly selected as the opposite.

A second reason for why we might not observe an effect of selection for codon bias on patterns of protein evolution could be because many non-synonymous polymorphisms are subject to strong natural selection, either positive or negative, and hence the additional selection pressure associated with selection on codon usage, which may be quite weak (Sharp et al. 2010), is irrelevant. Levels of non-synonymous relative to synonymous polymorphism are typically very low in bacteria (Hughes et al. 2008), consistent with strong selection against non-synonymous mutations.

Finally, it is possible that all codons of some amino acids are more efficiently translated than others due to, for example, the concentrations for all tRNAs matching some codons being greater than others. If this was the case, then this would mask an effect. However, tRNA concentrations are similar for all amino acids in *E. coli* (Ikemura 1981) and it is likely that this is the optimal strategy for a cell—i.e. we wouldn’t expect the system to evolve so that some amino acids were translated much more efficiently than others.

Overall, we found no evidence for an impact of selection on codon usage on the rates of protein evolution in bacteria.

## Materials and Methods

### Population Genomic Data and Data Filtering

Polymorphism data were downloaded from the ATGC database (Kristensen et al. 2017), where full orthologous genome alignments were obtained for *E. coli* (ATGC001) and *S. pneumoniae* (ATGC003). We filtered the data to keep only genes present in the core genome, resulting in a total of 1,316 and 1,194 genes in 164 and 31 strains of *E. coli* and *S. pneumoniae*, respectively. These alignments were then split by gene and codon statistics were obtained with the BppPopStats program (Guéguen et al. 2013).

### Gene Expression

For *E. coli*, we used the compiled gene expression data set from the DREAM5 network inference challenge (Marbach et al. 2012), resulting in a total of 1,287 genes for analysis. For *S. pneumoniae*, gene expression data were obtained from the Gene Expression Omnibus (GEO) database (Edgar et al. 2002) where we used the normalized data set GSE77587, ending up with 803 genes for further analysis. Analyses were performed by splitting the data into five categories (one being the lowest—∼8.10, and five being the highest—∼12.05—units in log RPKM) of gene expression with similar number of genes in each category.

### Correspondence Analysis and RSCU Estimates

To identify the preferred codons, we performed correspondence analyses on the two species using the strains *E. coli K12 MG1655* and *S. pneumoniae R6* with the complete set of core genes (1,316 and 1,194 for *E. coli* and *S. pneumoniae*, respectively). The absolute frequency of codons (AF) and the RSCU were estimated using the function “uco” from the “seqinr” R package (Charif and Lobry 2007) for the full set of genes. The correspondence analyses (CA) were performed with the function “dudi.coa” from the “ade4” R package (Dray and Dufour 2007) using the RSCU (CA-RSCU) and AF values (CA-AF). The within-group correspondence analyses (WCA) were performed using CA-AF (WCA-CA-AF). We compared the two methods (CA-RSCU and WCA-CA-AF) to check whether there were significant differences between them. The first two principal components were used to assess the amount of variation in CUB across genes. We identified genes showing a greater bias in synonymous codon usage by looking at the distribution of ribosomal proteins and other highly expressed genes on PC1. RSCU values of highly expressed genes were then estimated for the set of genes belonging to the 90% percentile of this distribution (i.e. the top 10%). The relative adaptiveness of each codon (*w*) and the CAI were estimated using these RSCU values as in Sharp and Li (1987a, 1987b). For the combined analyses with gene expression and CAI, RSCU values were separately estimated for the codons belonging to each category of each variable analyzed.

### The log(*Y*) Statistic

At the polymorphism level, we filtered the data to keep only sites containing at least 95% of the data (i.e. data in at least 95% of the strains to minimize the amount of missing data). We reconstructed the ancestral state using two different outgroups for each species to check for differences between them: *E. albertii* and *E. fergusonii* for *E. coli*, and *S. pseudopneumoniae* and *S. mitis* for *S. pneumoniae.* These were used to polarize each polymorphism. The synonymous genetic divergence (*d_S_*) for each species comparison was performed by dividing the number of synonymous substitutions by the number of synonymous sites at divergence (see supplementary File S1, Supplementary Material online). We only considered biallelic sites where the ancestral and derived alleles are separated by a single non-synonymous mutation (for example, Gln → Lys), resulting in a total of 129 amino acid non-synonymous mutations (out of 375 and 358 in *E. coli* and *S. pneumoniae*, respectively; after removing all amino acid mutations including Met and Trp, as these amino acids have a single codon each), including 381 (out of 2,153) and 379 (out of 1,841) codon mutations for analysis in *E. coli* and *S. pneumoniae*, respectively. The numbers differ slightly between the two species because for a few amino acid pairs, there were no data. As RSCU values have different maximum values in the presence of selection (i.e. two for a 2-fold and four for a 4-fold), amino acids with different degeneracies are not comparable. We therefore divided our analyses into comparisons between amino acids where both amino acids are 2-fold degenerate and comparisons in which both amino acids are 4-fold degenerate. This analysis was performed with 30 and 12 amino acid mutations in each species, for 2-folds and 4-folds, respectively. The number of polymorphisms used in the analysis relative to Figs. 1 and 2 can be found in supplementary table S1, Supplementary Material online.

At the divergence level, we polarized codon substitutions according to the two outgroups in each species (*E. albertii* and *E. fergusonii* for *E. coli*, and *S. pseudopneumoniae* and *S. mitis* for *S. pneumoniae*), analyzing only sites where the two outgroups matched. We considered only fixed sites within the population separated by one nucleotide substitution relative to the outgroups, resulting in a total of 79 (out of 296) and 120 (out of 379) amino acid substitutions in *E. coli* and *S. pneumoniae*, respectively. However, as amino acids with different degeneracies are not comparable, our final analyses included 20 and 8 amino acid substitutions for 2-fold and 4-fold, respectively, in *E. coli*, and 28 and 10 amino acid substitutions for 2-fold and 4-fold, respectively, in *S. pneumoniae*; log(*Y*_div_) was undefined for some amino acid combinations (the numbers of codon substitutions and fixed sites can be found in supplementary table S2, Supplementary Material online).

### Statistical Analysis

Statistical significance was assessed with Spearman's correlation coefficient. All figures were plotted using the R package “ggplot2” (Wickham 2017). Statistical significance for the combined effect of gene expression and CAI with log(*Y*_pol_), $\log(Y_{\mathrm{div}})$, and log(Δ*RSCU*) was assessed with a linear model. A *t*-test was used to assess whether the means of the distribution of log(*Y*_pol_) and $\log(Y_{\mathrm{div}})$ were significantly different from zero.

### Power Analyses

We investigated the power of our analysis using two related approaches. We focused our attention on the log(*Y*_pol_) because this is the analysis for which we have the most data—we have more polymorphism data per amino acid comparison and we are not splitting the data—and this analysis addresses the fundamental question directly—does selection on codon usage affect patterns of protein evolution? In the first analysis, we estimated the probability of detecting an effect. Let us imagine that the expected value of *Y*_pol_ is $\hat{Y}$ then we calculate the expected number of polymorphisms increasing RSCU as $(1+\alpha )\bar{p}N_{\mathrm{low}}$ and the expected number decreasing them as $(1-\alpha )\bar{p}N_{\mathrm{high}}$ where $\alpha =(\hat{Y}-1)/(\hat{Y}+1)$, *p* is the number of polymorphisms per site for the pair of amino acids being considered (e.g. Gln → Lys), and *N*_low_ and *N*_high_ are the numbers of codons that have the high and low RSCU value; for Gln, these would be CAA and CAG, respectively. For every amino acid pair, we calculate these expected values and generate a random Poisson variate to recalculate the log(*Y*_pol_) values. We then performed a *t*-test to test whether these simulated log(*Y*_pol_) values were significantly greater than 0. This was repeated 10,000 times.

It is also possible to estimate the expected value of log(*Y*_pol_) as follows. Let us assume that there is no mutation bias and that CUB is determined by a balance between mutation, selection, and genetic drift (Li 1987 JME). Consider a 2-fold degenerate codon. Let the preferred codon, A2, have an advantage of *s* over the unpreferred codon, A1. If the mutation rate, *u*, is such that *N_e_u* ≪ 1 then the proportion of sites fixed for the A2 allele is:


(5) $$f=\frac{e^{S}}{e^{S}+1}$$

where *S* = 4*N_e_s* (Li 1987). For this equation, it is possible to estimate *S* from the proportion of sites that are A2 compared to A1 in a genome sequence. In our analysis, we have two amino acids connected to a single non-synonymous mutation; let *S*_ancestral_ be the selection strength associated with codon usage in the ancestral amino acid (Gln in the case of Gln → Lys) and *S*_derived_ be the strength of selection for the derived amino acid (Lys). Let us assume that there is no selection upon the amino acid change itself then a mutation from the ancestral to the derived state involves a strength of selection in favor of increasing the RSCU of *S*_derived_ and selection against decreasing RSCU of −*S*_ancestral_. Using these strengths of selection, we can estimate the number of polymorphisms that increase and decrease RSCU and hence the expected value of log(*Y*). If *S* is the strength of selection in favor of a mutation, multiplied by *N_e_*, then the expected number of polymorphisms in a sample of *n* chromosomes is:


(6) $$P=\int_{0}^{\text{1}}\frac{1-e^{-2S(1-x)}}{x(1-x)(1-e^{-2S})}(1-x^{n}-{\left(1-x\right)}^{n})dx$$

(Wright 1938).

## Supplementary Material

Supplementary material is available at *Genome Biology and Evolution* online.

## Supplementary Material

evad232_Supplementary_Data

## Data Availability

All data and code necessary to reproduce these analyses are available at https://figshare.com/s/bbf342720f88860c5aef.
