# Supplementary material for: No Evidence that Selection on Synonymous Codon Usage Affects Patterns of Protein Evolution in Bacteria
Source: Genome Biol Evol. 2023 Dec 27;16(2):evad232. doi: 10.1093/gbe/evad232 (PMC10849182; doi:10.1093/gbe/evad232)
Supplement: evad232_Supplementary_Data [file evad232_supplementary_data.zip › FileS2.pdf]

Divergence analysis

af_moutinho

2022-07-04

This RMarkdown contains all analyses performed at the divergence level. The first chunk of the script reproduces the estimation of Z in *E. coli* and *S. pneumoniae* using the RSCU values of highly expressed genes.

```
## Libraries
library(plyr)
library(dplyr)
library(data.table)
library(ggplot2)
library(ggpubr)
library(kableExtra)
library(RGenetics)
library(cowplot)
library(tidyr)
library(stringi)
library(stringr)
library(Hmisc)
library(RGenetics)
library(seqinr)
##

## adding the font needed for the plot
library(showtext)
font_add_google("Noto Serif")
showtext_auto()
##

## table containing all possible codon pairs for E. coli and S. pneumoniae
## using the RSCU value for highly expressed genes
## change path to where you keep the data
codons_poly_main <- fread(file = "~/Dropbox/CUB_supplementary_data/tables/polymorphims_tbl_main.csv",
                          sep = "\t", header = T)
codons_div <- subset(codons_poly_main, select = - c(MeanNumberSynPos, Is4Degenerated, IsSynPoly, PiN, Pi
                                                    Freq, rscu_derived, deg_derived, aa_derived, nt.dif
                                                    fold, DerivedAllele, MissingDataFrequency, aa_ances
                                                    rscu_ancestral, deg_ancestral, deg_derived))
colnames(codons_div) <- gsub("OutgroupAllele", "out1", colnames(codons_div))

codons_poly_all <- fread(file = "~/Dropbox/CUB_supplementary_data/tables/polymorphims_tbl_all.csv",
                         sep = "\t", header = T)

## estimating dS for each species pair
```

```
dS_df <- ddply(codons_poly_all, c("species", "outgroup"), function(x) {
  dS <- sum(x$dS, na.rm = T)/sum(x$MeanNumberSynPosDiv, na.rm = T)
  data.frame(dS)
})

## getting the other outgroups (distantly-related)
outs <- subset(codons_poly_all, outgroup == "efergusonii" | outgroup == "smitis")
outs_df <- subset(outs, select = c("OutgroupAllele", "Site", "coID", "species", "MajorAllele", "MinorAl
colnames(outs_df)[c(1,7,8)] <- c("out2", "dN2", "MeanNumberSynPosDiv2")

## combining the two tables:
codons_div <- left_join(codons_div, outs_df, by = c("Site", "coID", "species", "MajorAllele", "MinorAll
head(codons_div)
```

AncestralAllele Site NbAlleles MinorAlleleFrequency MajorAlleleFrequency 1: AAA 446 1 164 164 2: AAA 142 1 164 164 3: AAA 35 1 164 164 4: AAA 191 1 164 164 5: AAA 324 1 164 164 6: AAA 70 1 164 164 MinorAllele MajorAllele out1 MeanNumberSynPosDiv dN dS coID 1: AAA AAA AAA 0.687979 0 0 ATGC001.COG01238 2: AAA AAA AAG 0.597236 0 1 ATGC001.COG02040 3: AAA AAA AAA 0.709052 0 0 ATGC001.COG00596 4: AAA AAA AAA 0.720643 0 0 ATGC001.COG01125 5: AAA AAA AAA 0.663397 0 0 ATGC001.COG00248 6: AAA AAA AAA 0.999983 0 0 ATGC001.COG00730 species cai_major mean_exp out2 dN2 MeanNumberSynPosDiv2 1: ecoli 0.5221362 9.396455 AAA 0 0.615619 2: ecoli 0.4393080 8.939308 AAG 0 0.572717 3: ecoli 0.5385140 9.898442 AAA 0 0.629626 4: ecoli 0.5725322 10.012101 AAA 0 0.665945 5: ecoli 0.4871850 9.182809 AAG 0 0.542325 6: ecoli 0.5300002 NA AAA 0 0.999983

```
## adding amino acids and degneracy:
df_genetic_code <- geneticCodeTable(DNA = T)
df_genetic_code <- subset(df_genetic_code, select = c("GeneticCode", "AminoAcids"))
df_genetic_code <- ddply(df_genetic_code, "AminoAcids", function(x) {
  x$deg <- nrow(x)
  return(x)
})
colnames(df_genetic_code) <- c("MajorAllele", "aa_major", "deg_major")
codons_div <- left_join(codons_div, df_genetic_code, by = "MajorAllele")
colnames(df_genetic_code) <- c("out1", "aa_out", "deg_out")
codons_div <- left_join(codons_div, df_genetic_code, by = "out1")

## adding the fold:
# out
codons_div$fold[codons_div$deg_major == 2 & codons_div$deg_out == 2] <- "2-fold"
codons_div$fold[codons_div$deg_major == 4 & codons_div$deg_out == 4] <- "4-fold"

## keeping only monomorphic sites:
mono_sites_df <- codons_div[codons_div$NbAlleles == 1,]
mono_sites_df$aa_mut <- paste0(mono_sites_df$aa_out, ">", mono_sites_df$aa_major)

## counting the number of sites that are the same between ingroup and outgroup
# both ingroup = out1 = out2
mono_sites_counts <- ddply(mono_sites_df, c("species", "MajorAllele"), function(x) {
  N <- nrow(x[x$MajorAllele == x$out1 & x$MajorAllele == x$out2,])
  data.frame(N)
})
```

```
## keeping only the sites with non-syn fixed differences:
ns_substitution_sites <- mono_sites_df[with(mono_sites_df, out1 == out2 &
                                aa_major != aa_out),]

## adding the codon and and amino acid mutation:
# both
ns_substitution_sites$codon_mut <- paste0(ns_substitution_sites$out1, ">", ns_substitution_sites$MajorAl[illegible]
ns_substitution_sites$aa_mut <- paste0(ns_substitution_sites$aa_out, ">", ns_substitution_sites$aa_majo[illegible]

## counting the number of fixed differences between ingroup and outgroup ingroup != (out1 = out2):
fixed_sites_counts <- ddply(ns_substitution_sites, c("species", "aa_mut", "codon_mut", "MajorAllele", "[illegible]
                            function(x) {
  n_subs <- nrow(x)
  data.frame(n_subs)
})


## combining the two tables:
subs_counts_df <- left_join(fixed_sites_counts, mono_sites_counts, by = c("species", "MajorAllele"))

## adding RSCU values:
uco_all <- read.table(file = "~/Dropbox/CUB_supplementary_data/tables/uco_all_df.csv",
                      sep = "\t", header = T)
# major allele
uco_major <- subset(uco_all, select = c("aa", "codon", "species", "rscu_major"))
colnames(uco_major)[c(1,2)] <- c("aa_major", "MajorAllele")
subs_counts_df <- left_join(subs_counts_df, uco_major, by = c("species", "MajorAllele"))
# outgroup:
uco_out <- subset(uco_all, select = c("aa", "codon", "species", "rscu_out"))
colnames(uco_out)[c(1,2)] <- c("aa_out", "out1")
subs_counts_df <- left_join(subs_counts_df, uco_out, by = c("species", "out1"))


## function to add nucleotide differences between codons:
string.diff.ex <- function(a="ATTCGAN",b="attTGTT",exclude=c("n","N","?"),ignore.case=TRUE)
{
  if(nchar(a)!=nchar(b)) stop("Lengths of input strings differ. Please check your input.")
  if(ignore.case==TRUE)
  {
    a<-toupper(a)
    b<-toupper(b)
  }
  diff.a<-unlist(strsplit(a,split=""))
  diff.b<-unlist(strsplit(b,split=""))
  diff.d<-rbind(diff.a,diff.b)
  for(ex.loop in 1:length(exclude))
  {
    diff.d<-diff.d[,!(diff.d[1,]==exclude[ex.loop]|diff.d[2,]==exclude[ex.loop])]
  }
  differences<-sum(diff.d[1,]!=diff.d[2,])
  return(differences)
}
```

```
## applying the function between codons:
subs_counts_df <- ddply(subs_counts_df, c("species", "out1", "MajorAllele"),
                        function(y) {
                          y$nt.dif <- string.diff.ex(unique(y$out1), unique(y$MajorAllele))
                          return(y)
                          })


write.table(subs_counts_df, file = "~/Dropbox/CUB_supplementary_data/tables/TableS2.csv",
            sep = "\t", col.names = T, row.names = F, quote = F)

### getting the tables and estimating log(Y)_div
#subs_counts_df <- read.table(file = "~/Dropbox/CUB_supplementary_data/tables/Revision3/codons_subs_tbl
#                              sep = "\t", header = T)

## keeping only codon pairs with 1 nucleotide difference and only those involving
## non-synonymous substitutions:
ns_codon_subs <- subset(subs_counts_df, nt.dif == 1)

## estimating the weighted averages:
## NOTE: adding 1e-04 to also consider codons where RSCU is 0
ns_codon_subs$log_rscu <- with(ns_codon_subs, round(log((rscu_major + 1e-04)/(rscu_out + 1e-04)), digit

## estimating Z as log(Y):
## both outgroups:
logY_div <- ddply(ns_codon_subs, c("species", "aa_mut", "aa_out", "aa_major", "fold"),
              function(x) {
                # maximum increase in RSCU
                max_rscu <- which.max(x$log_rscu)
                # maximum decrease in RSCU
                min_rscu <- which.min(x$log_rscu)
                # log(Y)
                logY_div <- log((x$n_subs[max_rscu]*x$N[min_rscu])/(x$n_subs[min_rscu]*x$N[max_rscu]))
                # log(deltaRSCU)
                log_rscu <- round(log(((x$rscu_major[max_rscu] + 1e-04)/(x$rscu_out[max_rscu] + 1e-04))
                               ((x$rscu_major[min_rscu] +  1e-04)/(x$rscu_out[min_rscu] + 1e-04))), dig
                data.frame(logY_div, log_rscu)
              })


## keeping only 2 and 4-folds
logY_div2 <- logY_div[is.na(logY_div$fold) == F,]
logY_div2$species <- factor(logY_div2$species,
                                   levels = c("ecoli", "spneumoniae"))
levels(logY_div2$species) <- c(expression(italic("E. coli")),
                                      expression(italic("S. pneumoniae")))

## plotting the distribution of Z values:
plot_dist_logY_div <- ggplot(logY_div2, aes(x=logY_div)) +
  geom_histogram(aes(y=..count..),
                 binwidth=.2,
                 colour="black", fill="white") +
  xlab(expression(atop(italic(log(Y[div]))))) +
```

```
  ylab("Frequency") +
  geom_vline(data = ddply(logY_div2, c("species", "fold"), summarise, avg = mean(logY_div)),
             aes(xintercept=avg),linetype="dashed",
             color = "azure4", size=0.5) +
  geom_text(data = ddply(logY_div2, c("species", "fold"), summarise, avg = mean(logY_div)),
            aes(x = -4, y = 2.5, label = paste("mean(log(Y)_div) = ", round(avg, digits = 3), sep = ""))
            hjust = 0,family = "Noto Serif", size = 4, color = "azure4") +
  theme_bw() +
  facet_wrap(fold~species, scales = "free", labeller = function(x) {label_parsed(x[2])}) +
  theme(text = element_text(family = "Noto Serif", size = 14),
        axis.text = element_text(family = "Noto Serif", size = 14),
        strip.text.x = element_text(family = "Noto Serif", size = 14),
        strip.text.y = element_text(family = "Noto Serif", face = "bold", size = 14),
        strip.background.x = element_rect(fill = "gray90", linetype = "blank"),
        strip.background.y = element_rect(fill = "gray90", linetype = "blank"))
plot_dist_logY_div
```

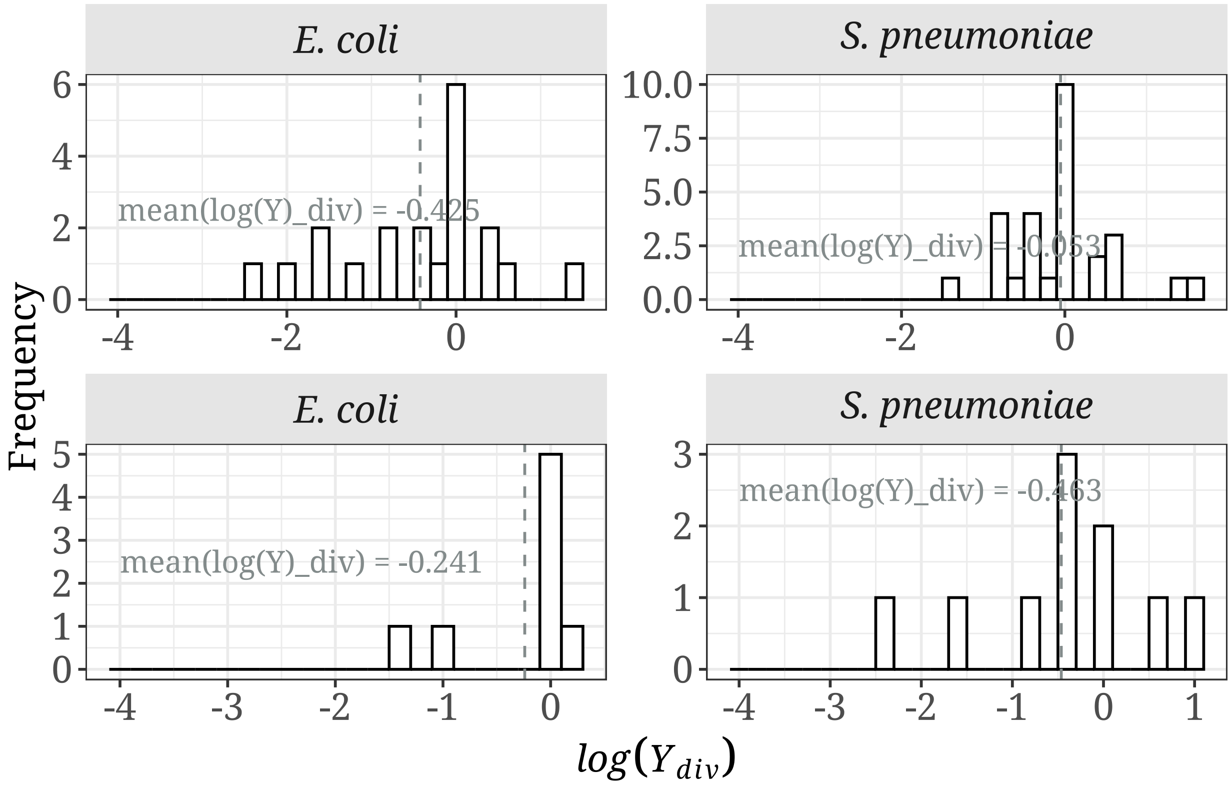


```
ggsave(filename = "Figure3.pdf", plot = plot_dist_logY_div, device = "pdf",
       path = "~/Dropbox/CUB_supplementary_data/Figures/", width = 8, height = 7, units = "in")

# checking if the mean is different from 0:
logY_div_prob <- ddply(logY_div2, c("species", "fold"), function(x) {
  t_test <- t.test(x$logY_div)
  mean <- t_test$estimate
  p_value <- t_test$p.value
```

```
  data.frame(mean, p_value)
})
kable(logY_div_prob)
```

species	fold	mean	p_value
italic("E. coli")	2-fold	-0.4245510	0.0568262
italic("E. coli")	4-fold	-0.2405765	0.2700159
italic("S. pneumoniae")	2-fold	-0.0525072	0.6766435
italic("S. pneumoniae")	4-fold	-0.4634015	0.1675780

```
## all analysis suggest that non synonymous substitutions are detrimental --> they reduce the RSCU valu
```

The next chunk of the script contains the analysis of the relationship between *log(Y)_div* and the log of the difference in RSCU:

```
## log(Y)_div ~ log(rscu)
## both outs:

plot_logY_div_rscu <- ggplot(logY_div2, aes(log_rscu, logY_div)) +
  geom_point(size = 0.6) +
  geom_smooth(method = "lm", formula = y~x, se = T, color = "black", size = 1) +
  xlab(expression(paste("log(", italic(Delta*RSCU), ")", sep = ""))) +
  ylab(expression(atop(italic(log(Y[div]))))) +
  #xlab(expression(paste("log(", italic(Delta*RSCU), ")", sep = ""))) +
  stat_cor(label.x.npc = "left", label.y.npc = "top", method = "spearman", size = 3.5,
           cor.coef.name = "rho") +
  facet_wrap(fold~species, scales = "free", labeller = function(x) {label_parsed(x[2])}) +
  theme_bw() +
  theme(text = element_text(family = "Noto Serif", size = 14),
        strip.text.x = element_text(family = "Noto Serif", size = 14),
        strip.text.y = element_text(family = "Noto Serif", face = "bold", size = 14),
        strip.background.x = element_rect(fill = "gray90", linetype = "blank"),
        strip.background.y = element_rect(fill = "gray90", linetype = "blank"))
plot_logY_div_rscu
```

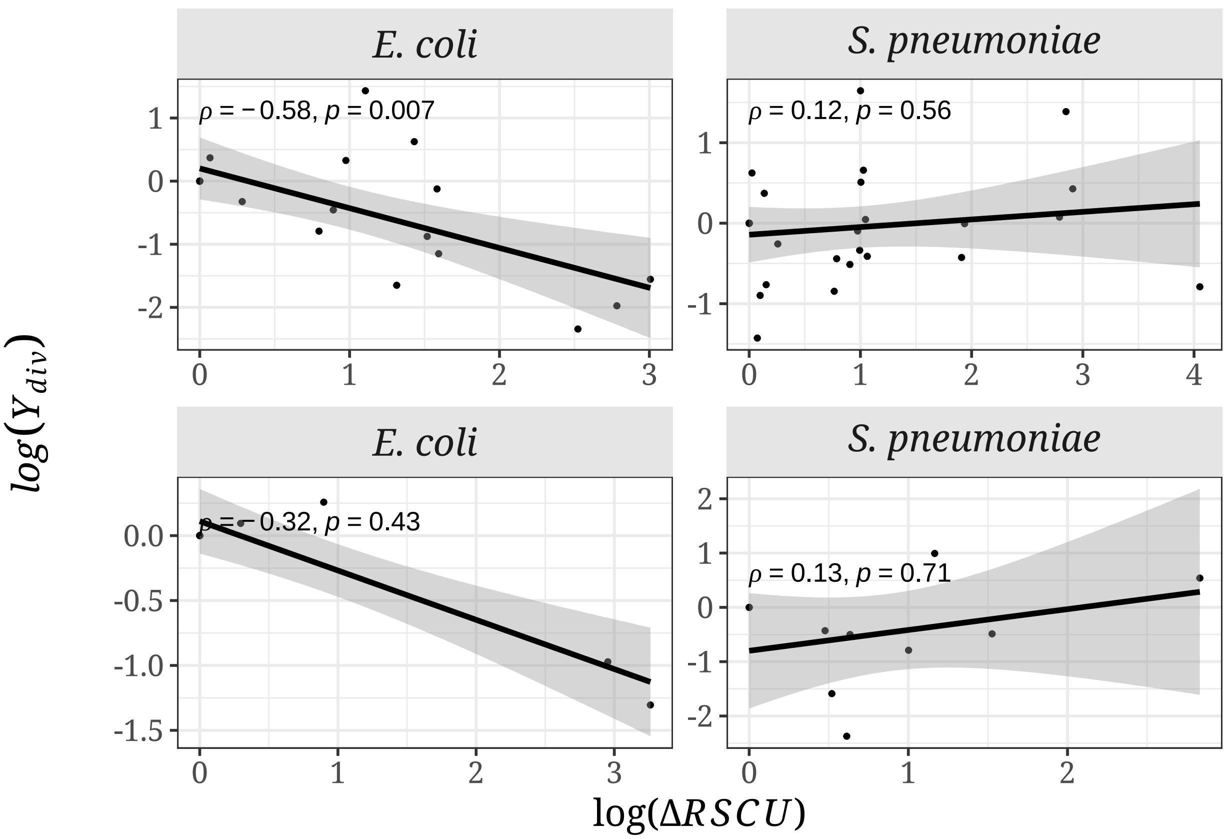


```
ggsave(filename = "Figure4.pdf", plot = plot_logY_div_rscu, device = "pdf",
       path = "~/Dropbox/CUB_supplementary_data/Figures/",
       width = 8.13, height = 6.42, units = "in")
```

Analysis of *log(Y)_div* taking into account CAI

```
## estimating RSCU values for each expression category
codons_div$cai_cat <- as.numeric(cut2(codons_div$cai_major, g = 3))

cai_df <- subset(codons_div, is.na(cai_cat) == F)

## getting mean expression levels for each category:
mean_cai_df <- ddply(cai_df, c("species", "cai_cat"), function(x) {
  mean_cai <- mean(x$cai_major)
  data.frame(mean_cai)
})

## split data.frame into each expression category in each species
splits_df <- cai_df %>%
  split(list(cai_df$species, cai_df$cai_cat), drop = T)


## get all codons per expression category (taking the major allele)
## MajorAllele
codon_cai_major <- lapply(splits_df, function(x) {
```

```
  unlist(str_split(x$MajorAllele, ""))
})

## get rscu per codon for each category:
uco_cai_major <- list()
for (i in 1:length(codon_cai_major)) {
  uco_cai_major[[i]] <- uco(codon_cai_major[[i]], as.data.frame = TRUE)
  uco_cai_major[[i]]$species <- str_split_fixed(names(codon_cai_major[i]), "\\.", 2)[,1]
  uco_cai_major[[i]]$cai_cat <- as.numeric(str_split_fixed(names(codon_cai_major[i]), "\\.", 2)[,2])
}

## combining all tables:
df_uco_cai_major <- rbindlist(uco_cai_major)

## keeping only columns of interest:
df_uco_cai_major <- subset(df_uco_cai_major, select = - c(eff, freq))
df_uco_cai_major$codon <- toupper(df_uco_cai_major$codon)

## Outgroup Allele
codon_cai_out <- lapply(splits_df, function(x) {
  unlist(str_split(x$out1, ""))
})

## get rscu per codon for each category:
uco_cai_out <- list()
for (i in 1:length(codon_cai_out)) {
  uco_cai_out[[i]] <- uco(codon_cai_out[[i]], as.data.frame = TRUE)
  uco_cai_out[[i]]$species <- str_split_fixed(names(codon_cai_out[i]), "\\.", 2)[,1]
  uco_cai_out[[i]]$cai_cat <- as.numeric(str_split_fixed(names(codon_cai_out[i]), "\\.", 2)[,2])
}

## combining all tables:
df_uco_cai_out <- rbindlist(uco_cai_out)

## keeping only columns of interest:
df_uco_cai_out <- subset(df_uco_cai_out, select = - c(eff, freq))
df_uco_cai_out$codon <- toupper(df_uco_cai_out$codon)


## keeping ony monomorphic sites:
mono_sites_cai <- subset(cai_df, NbAlleles == 1)

## counting mono sites:
mono_counts_cai <- subset(mono_sites_cai, MajorAllele == out1 & MajorAllele == out2)
mono_counts_cai2 <- ddply(mono_counts_cai, c("species", "cai_cat", "MajorAllele"), function(x) {
  N <- nrow(x)
  data.frame(N)
})

## doing the counts of each substitution in each exp category:
subs_counts_cai <- subset(mono_sites_cai, out1 == out2 & MajorAllele != out1)
subs_counts_cai2 <- ddply(subs_counts_cai, c("species", "cai_cat", "MajorAllele", "out1"), function(x) 
  n_subs <- nrow(x)
```

```
  data.frame(n_subs)
})

## combining with the table with all the codon combinations:
codons_cai_df <- left_join(subs_counts_cai2, mono_counts_cai2, by = c("species", "cai_cat", "MajorAllele

## adding RSCU values to the main table
colnames(df_uco_cai_major)[c(1:3)] <- c("aa_major", "MajorAllele", "rscu_major")
codons_cai_df <- left_join(codons_cai_df, df_uco_cai_major, by = c("MajorAllele", "species", "cai_cat")

colnames(df_uco_cai_out)[c(1:3)] <- c("aa_out", "out1", "rscu_out")
codons_cai_df <- left_join(codons_cai_df, df_uco_cai_out, by = c("out1", "species", "cai_cat"))

## getting the nucleotide differences between codons:
codons_cai_df2 <- ddply(codons_cai_df, c("species", "cai_cat", "MajorAllele", "out1"), function(y) {
                         y$nt.dif <- string.diff.ex(unique(y$MajorAllele),
                                                    unique(y$out1))
                         return(y)
                       })

## taking only non-syn mutations separated by 1 mutational step:
ns_codons_cai <- subset(codons_cai_df2, nt.dif == 1 & aa_major != aa_out)

## adding aa pairs:
ns_codons_cai$codon_mut <- paste0(ns_codons_cai$out1, ">", ns_codons_cai$MajorAllele)
ns_codons_cai$aa_mut <- paste0(ns_codons_cai$aa_out, ">", ns_codons_cai$aa_major)

## estimating the weighted averages:
## estimating the weighted averages:
## NOTE: adding 1e-04 to also consider codons where RSCU is 0
ns_codons_cai$log_rscu <- with(ns_codons_cai, round(log((rscu_major + 1e-04)/(rscu_out + 1e-04)), digit

## adding degeneracy
head(df_genetic_code)
```

```
##   out1 aa_out deg_out
## 1  GCA    Ala       4
## 2  GCT    Ala       4
## 3  GCC    Ala       4
## 4  GCG    Ala       4
## 5  AGA    Arg       6
## 6  AGG    Arg       6
```

```
colnames(df_genetic_code) <- c("out1", "aa_out", "deg_out")
ns_codons_cai <- left_join(ns_codons_cai, df_genetic_code, by = c("out1", "aa_out"))
colnames(df_genetic_code) <- c("MajorAllele", "aa_major", "deg_major")
ns_codons_cai <- left_join(ns_codons_cai, df_genetic_code, by = c("MajorAllele", "aa_major"))

ns_codons_cai$fold[ns_codons_cai$deg_major == 2 & ns_codons_cai$deg_out == 2] <- "2-fold"
ns_codons_cai$fold[ns_codons_cai$deg_major == 4 & ns_codons_cai$deg_out == 4] <- "4-fold"
# removing the amino acid pairs that are not 2-fold or 4-fold
ns_codons_cai <- ns_codons_cai[is.na(ns_codons_cai$fold) == F,]
write.csv(ns_codons_cai, file = "~/Dropbox/CUB_supplementary_data/tables/TableS5.csv",
```

```r
          row.names = F, quote = F)

## estimating Z as log(Y):
## both outgroups:
logY_div_cai <- ddply(ns_codons_cai, c("species", "cai_cat", "aa_mut", "aa_out", "aa_major", "fold"),
              function(x) {
                # maximum increase in RSCU
                max_rscu <- which.max(x$log_rscu)
                # maximum decrease in RSCU
                min_rscu <- which.min(x$log_rscu)
                # log(Y)
                logY_div <- log((x$n_subs[max_rscu]*x$N[min_rscu])/(x$n_subs[min_rscu]*x$N[max_rscu]))
                # log(deltaRSCU)
                log_rscu <- round(log(((x$rscu_major[max_rscu] + 1e-04)/(x$rscu_out[max_rscu] + 1e-04)),
                               ((x$rscu_major[min_rscu] +  1e-04)/(x$rscu_out[min_rscu] + 1e-04))), dig
                data.frame(logY_div, log_rscu)
              })

# adding the mean exp:
logY_div_cai <- left_join(logY_div_cai, mean_cai_df, by = c("species", "cai_cat"))

## keeping only 2 and 4-folds
logY_div_cai2 <- logY_div_cai[is.na(logY_div_cai$fold) == F,]
logY_div_cai2$species <- factor(logY_div_cai2$species,
                                levels = c("ecoli", "spneumoniae"))
levels(logY_div_cai2$species) <- c(expression(italic("E. coli")),
                                   expression(italic("S. pneumoniae")))
#write.table(sum_weights_exp2, file = "~/path-to/CUB_supplementary_data/tables/Z_exp.csv",
#             sep = "\t", col.names = T, row.names = F)


## log(Y)_div ~ log(rscu) for each gene expression category
p.RSCU.logY_div_cai <- ggplot(logY_div_cai2, aes(log_rscu, logY_div, label = aa_mut,
                                                  col=as.ordered(cai_cat))) +
  geom_point(size = 0.6) +
  geom_smooth(method = "glm", formula = y~x, se = F, size = 1) +
  xlab(expression(paste("log(", italic(Delta*RSCU), ")", sep = ""))) +
  ylab(expression(atop(italic(log(Y[div]))))) +
  guides(col=guide_legend(title="CAI")) +
  #facet_grid(fold~species, scales = "free", labeller = label_parsed) +
  facet_wrap(fold~species, scales = "free", labeller = function(x) {label_parsed(x[2])}) +
  theme_bw() +
  theme(text = element_text(family = "Noto Serif", size = 12),
        strip.text.x = element_text(family = "Noto Serif", size = 12),
        strip.text.y = element_text(family = "Noto Serif", face = "bold", size = 12),
        strip.background.x = element_rect(fill = "gray90", linetype = "blank"),
        strip.background.y = element_rect(fill = "gray90", linetype = "blank"))
p.RSCU.logY_div_cai
```

```
## Warning: The following aesthetics were dropped during statistical transformation: label
## i This can happen when ggplot fails to infer the correct grouping structure in
##   the data.
## i Did you forget to specify a `group` aesthetic or to convert a numerical
```

```
##   variable into a factor?
## The following aesthetics were dropped during statistical transformation: label
## i This can happen when ggplot fails to infer the correct grouping structure in
##   the data.
## i Did you forget to specify a ‘group‘ aesthetic or to convert a numerical
##   variable into a factor?
## The following aesthetics were dropped during statistical transformation: label
## i This can happen when ggplot fails to infer the correct grouping structure in
##   the data.
## i Did you forget to specify a ‘group‘ aesthetic or to convert a numerical
##   variable into a factor?
## The following aesthetics were dropped during statistical transformation: label
## i This can happen when ggplot fails to infer the correct grouping structure in
##   the data.
## i Did you forget to specify a ‘group‘ aesthetic or to convert a numerical
##   variable into a factor?
```

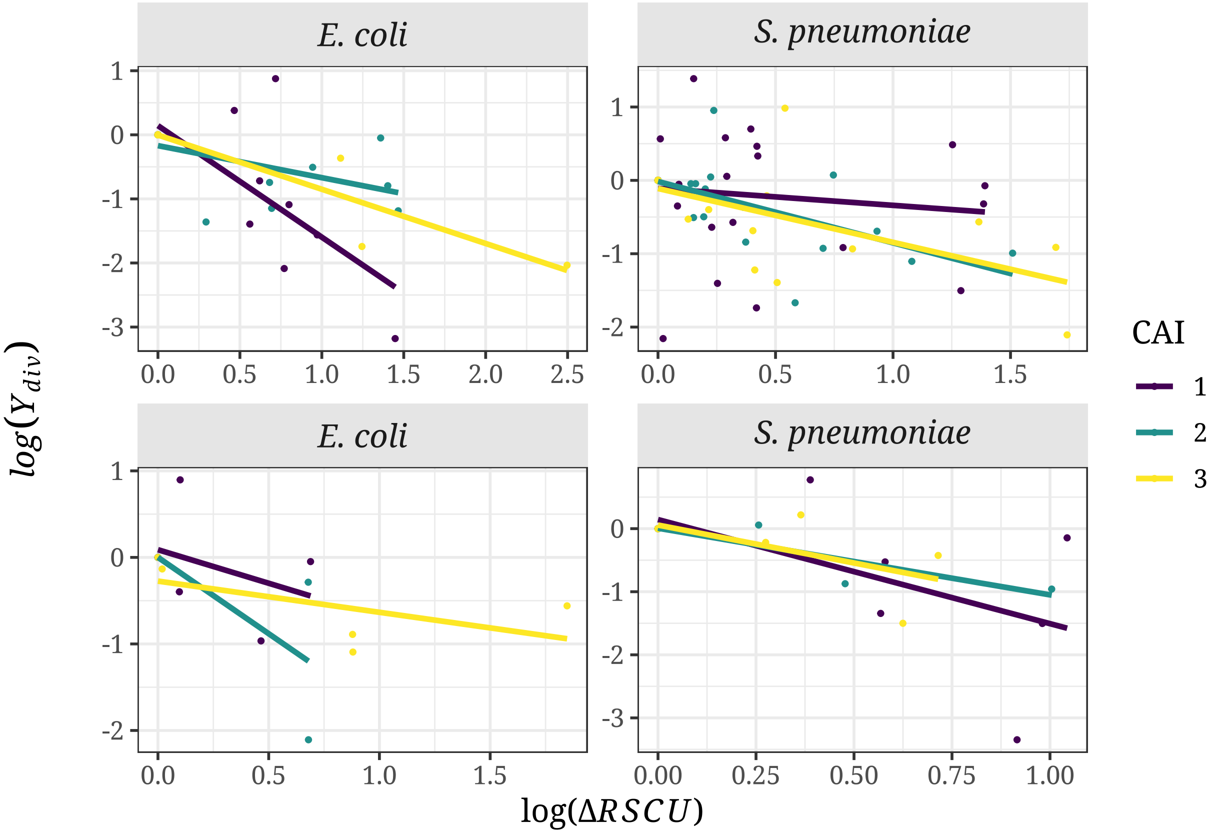


```
ggsave(filename = "Figure6.pdf", plot = p.RSCU.logY_div_cai, device = "pdf",
       path = "~/Dropbox/CUB_supplementary_data/Figures/", width = 8.84,
       height = 6.68, units = "in")
```

```
## Warning: The following aesthetics were dropped during statistical transformation: label
## i This can happen when ggplot fails to infer the correct grouping structure in
##   the data.
## i Did you forget to specify a ‘group‘ aesthetic or to convert a numerical
```

```
##   variable into a factor?
## The following aesthetics were dropped during statistical transformation: label
## i This can happen when ggplot fails to infer the correct grouping structure in
##   the data.
## i Did you forget to specify a `group` aesthetic or to convert a numerical
##   variable into a factor?
## The following aesthetics were dropped during statistical transformation: label
## i This can happen when ggplot fails to infer the correct grouping structure in
##   the data.
## i Did you forget to specify a `group` aesthetic or to convert a numerical
##   variable into a factor?
## The following aesthetics were dropped during statistical transformation: label
## i This can happen when ggplot fails to infer the correct grouping structure in
##   the data.
## i Did you forget to specify a `group` aesthetic or to convert a numerical
##   variable into a factor?
```

```
## linear regressions:
stats_div_cai <- ddply(logY_div_cai2, c("species", "fold", "cai_cat"), function(x) {
  cor_test <- cor.test(x$logY_div, x$log_rscu, method = "spearman", exact = FALSE)
  rho <- cor_test$estimate
  p_value <- cor_test$p.value
  N <- nrow(x)
  data.frame(rho, p_value, N)
})
kable(stats_div_cai)
```

species	fold	cai_cat	rho	p_value	N
italic("E. coli")	2-fold	1	-0.6308725	0.0087823	16
italic("E. coli")	2-fold	2	-0.8345865	0.0002050	14
italic("E. coli")	2-fold	3	-1.0000000	0.0000000	9
italic("E. coli")	4-fold	1	-0.4117647	0.4172603	6
italic("E. coli")	4-fold	2	-1.0000000	0.0000000	4
italic("E. coli")	4-fold	3	-0.7000000	0.1881204	5
italic("S. pneumoniae")	2-fold	1	-0.1397243	0.4869953	27
italic("S. pneumoniae")	2-fold	2	-0.5697248	0.0036586	24
italic("S. pneumoniae")	2-fold	3	-0.6875800	0.0022865	17
italic("S. pneumoniae")	4-fold	1	-0.6896552	0.0398243	9
italic("S. pneumoniae")	4-fold	2	-0.5058824	0.1646903	9
italic("S. pneumoniae")	4-fold	3	-0.5675676	0.1422650	8

```
## relationship between RSCU and log(Y)_div
## relationship becomes more negative for higher gene expression categories
## cannot do the divergence analysis with gene expression - not enough data
```

Linear models:

```
## doing the linear model accounting for standard error:
## will do the linear models only for 2-folds (4-folds have too little data points)
ecoli_logY_cai_2fold <- subset(logY_div_cai2, species == 'italic("E. coli")' & fold == "2-fold")
ecoli_logY_cai_4fold <- subset(logY_div_cai2, species == 'italic("E. coli")' & fold == "4-fold")
spneu_logY_cai_2fold <- subset(logY_div_cai2, species == 'italic("S. pneumoniae")' & fold == "2-fold")
spneu_logY_cai_4fold <- subset(logY_div_cai2, species == 'italic("S. pneumoniae")' & fold == "4-fold")
```

```r
## E. coli
## 2-folds
lm_ecoli_2fold <- with(ecoli_logY_cai_2fold, lm(logY_div ~ log_rscu + cai_cat))
lm_ecoli2_2fold <- with(ecoli_logY_cai_2fold, lm(logY_div ~ log_rscu * cai_cat))
AIC(lm_ecoli_2fold, lm_ecoli2_2fold) # best with interaction
```

```
##                 df      AIC
## lm_ecoli_2fold   4 77.00554
## lm_ecoli2_2fold  5 75.81219
```

```r
summary(lm_ecoli2_2fold)
```

```
##
## Call:
## lm(formula = logY_div ~ log_rscu * cai_cat)
##
## Residuals:
##      Min       1Q   Median       3Q      Max
## -1.24284 -0.02994  0.00293  0.03350  1.82256
##
## Coefficients:
##                  Estimate Std. Error t value Pr(>|t|)
## (Intercept)       0.05819    0.31130   0.187 0.852784
## log_rscu         -1.70100    0.47061  -3.614 0.000937 ***
## cai_cat          -0.03056    0.15101  -0.202 0.840778
## log_rscu:cai_cat  0.34397    0.19904   1.728 0.092780 .
## ---
## Signif. codes:  0 '***' 0.001 '**' 0.01 '*' 0.05 '.' 0.1 ' ' 1
##
## Residual standard error: 0.5939 on 35 degrees of freedom
## Multiple R-squared:  0.522,  Adjusted R-squared:  0.481
## F-statistic: 12.74 on 3 and 35 DF,  p-value: 8.771e-06
```

```r
# information in TableS1
#             Estimate Std. Error t value Pr(>|t|)
#(Intercept)        0.05819    0.31130   0.187 0.852784
#log_rscu         -1.70100    0.47061  -3.614 0.000937 ***
#cai_cat          -0.03056    0.15101  -0.202 0.840778
#log_rscu:cai_cat  0.34397    0.19904   1.728 0.092780 .

## 4-folds
lm_ecoli_4fold <- with(ecoli_logY_cai_4fold, lm(logY_div ~ log_rscu + cai_cat))
lm_ecoli2_4fold <- with(ecoli_logY_cai_4fold, lm(logY_div ~ log_rscu * cai_cat))
AIC(lm_ecoli_4fold, lm_ecoli2_4fold) # best without interaction
```

```
##                 df      AIC
## lm_ecoli_4fold   4 34.12411
## lm_ecoli2_4fold  5 34.79710
```

```r
summary(lm_ecoli_4fold)
```

```
##
## Call:
## lm(formula = logY_div ~ log_rscu + cai_cat)
##
## Residuals:
##     Min      1Q  Median      3Q     Max
## -1.5822 -0.2280  0.1062  0.2334  0.9999
##
## Coefficients:
##             Estimate Std. Error t value Pr(>|t|)
## (Intercept)  0.04478    0.41353   0.108    0.916
## log_rscu    -0.57021    0.35832  -1.591    0.138
## cai_cat     -0.09118    0.21450  -0.425    0.678
##
## Residual standard error: 0.6463 on 12 degrees of freedom
## Multiple R-squared:  0.2471, Adjusted R-squared:  0.1217
## F-statistic:  1.97 on 2 and 12 DF,  p-value: 0.1821
```

```r
#             Estimate Std. Error t value Pr(>|t|)
#(Intercept)   0.04478    0.41353   0.108    0.916
#log_rscu    -0.57021    0.35832  -1.591    0.138
#cai_cat     -0.09118    0.21450  -0.425    0.678


## S. pneumoniae
## 2-folds
lm_spneu_2fold <- with(spneu_logY_cai_2fold, lm(logY_div ~ log_rscu + cai_cat))
lm_spneu2_2fold <- with(spneu_logY_cai_2fold, lm(logY_div ~ log_rscu * cai_cat))
AIC(lm_spneu_2fold, lm_spneu2_2fold) # best without interaction
```

```
##                 df      AIC
## lm_spneu_2fold   4 136.2676
## lm_spneu2_2fold  5 136.4729
```

```r
summary(lm_spneu_2fold)
```

```
##
## Call:
## lm(formula = logY_div ~ log_rscu + cai_cat)
##
## Residuals:
##      Min       1Q   Median       3Q      Max
## -2.15211 -0.27243  0.09205  0.19889  1.48706
##
## Coefficients:
##             Estimate Std. Error t value Pr(>|t|)
## (Intercept)  0.10872    0.20103   0.541 0.590483
## log_rscu    -0.57344    0.16401  -3.496 0.000855 ***
## cai_cat     -0.10080    0.09795  -1.029 0.307217
```

```
## ---
## Signif. codes:  0 '***' 0.001 '**' 0.01 '*' 0.05 '.' 0.1 ' ' 1
##
## Residual standard error: 0.6356 on 65 degrees of freedom
## Multiple R-squared:  0.1787, Adjusted R-squared:  0.1534
## F-statistic: 7.071 on 2 and 65 DF,  p-value: 0.001665
```

```
# information in TableS1
#             Estimate Std. Error t value Pr(>|t|)
#(Intercept)   0.10872    0.20103   0.541 0.590483
#log_rscu    -0.57344    0.16401  -3.496 0.000855 ***
#cai_cat     -0.10080    0.09795  -1.029 0.307217

## 4-folds
lm_spneu_4fold <- with(spneu_logY_cai_4fold, lm(logY_div ~ log_rscu + cai_cat))
lm_spneu2_4fold <- with(spneu_logY_cai_4fold, lm(logY_div ~ log_rscu * cai_cat))
AIC(lm_spneu_4fold, lm_spneu2_4fold) # best without interaction
```

```
##                 df      AIC
## lm_spneu_4fold   4 56.10035
## lm_spneu2_4fold  5 57.71069
```

```
summary(lm_spneu_4fold)
```

```
##
## Call:
## lm(formula = logY_div ~ log_rscu + cai_cat)
##
## Residuals:
##     Min      1Q  Median      3Q     Max
## -2.0984 -0.1054 -0.0590  0.2071  1.2958
##
## Coefficients:
##             Estimate Std. Error t value Pr(>|t|)
## (Intercept) -0.03375    0.39168  -0.086 0.932081
## log_rscu    -1.37672    0.35997  -3.825 0.000869 ***
## cai_cat      0.04638    0.16420   0.282 0.780140
## ---
## Signif. codes:  0 '***' 0.001 '**' 0.01 '*' 0.05 '.' 0.1 ' ' 1
##
## Residual standard error: 0.6488 on 23 degrees of freedom
## Multiple R-squared:  0.4198, Adjusted R-squared:  0.3693
## F-statistic:  8.32 on 2 and 23 DF,  p-value: 0.001911
```

```
#             Estimate Std. Error t value Pr(>|t|)
#(Intercept)  -0.03375    0.39168  -0.086 0.932081
#log_rscu    -1.37672    0.35997  -3.825 0.000869 ***
#cai_cat      0.04638    0.16420   0.282 0.780140
```
