# Supplementary material for: No Evidence that Selection on Synonymous Codon Usage Affects Patterns of Protein Evolution in Bacteria
Source: Genome Biol Evol. 2023 Dec 27;16(2):evad232. doi: 10.1093/gbe/evad232 (PMC10849182; doi:10.1093/gbe/evad232)
Supplement: evad232_Supplementary_Data [file evad232_supplementary_data.zip › Supplementary_material_r4.pdf]

Supplementary Tables

Table S3. Linear models' estimates for the polymorphism and substitution analyses CAI. We used *$log(Y_{pol})$* and *$log(Y_{div})$* as response variables in the polymorphism and substitution analyses, respectively, and *$log(\Delta RSCU)$,* CAI category, and their interaction with the response variable as explanatory variables.

			E. coli		*S. pneumoniae*	
		**Variable**	***$log(Y_{pol})$***	***$log(Y_{div})$***	***$log(Y_{pol})$***	***$log(Y_{div})$***
CAI	**2-folds**	**Intercept**	0.048	0.058	-0.039	0.109
		**log(*ΔRSCU*)**	0.079	-1.701 (***)	0.638 (***)	-0.573 (***)
		**CAI**	0.017	-0.031	-0.011	-0.101
		**Interaction**	-	0.344	-	-
	**4-folds**	**Intercept**	-0.449	0.045	0.328	-0.034
		**log(*ΔRSCU*)**	0.094	-0.570	-0.374	-1.377 (***)
		**CAI**	0.195 (.)	-0.091	0.002	0.046
		**Interaction**	-	-	-	-

Note. For each variable, the correlation coefficients are shown with the respective significance (*P < 0.05; **P < 0.01; ***P < 0.001).

Supplementary Figures

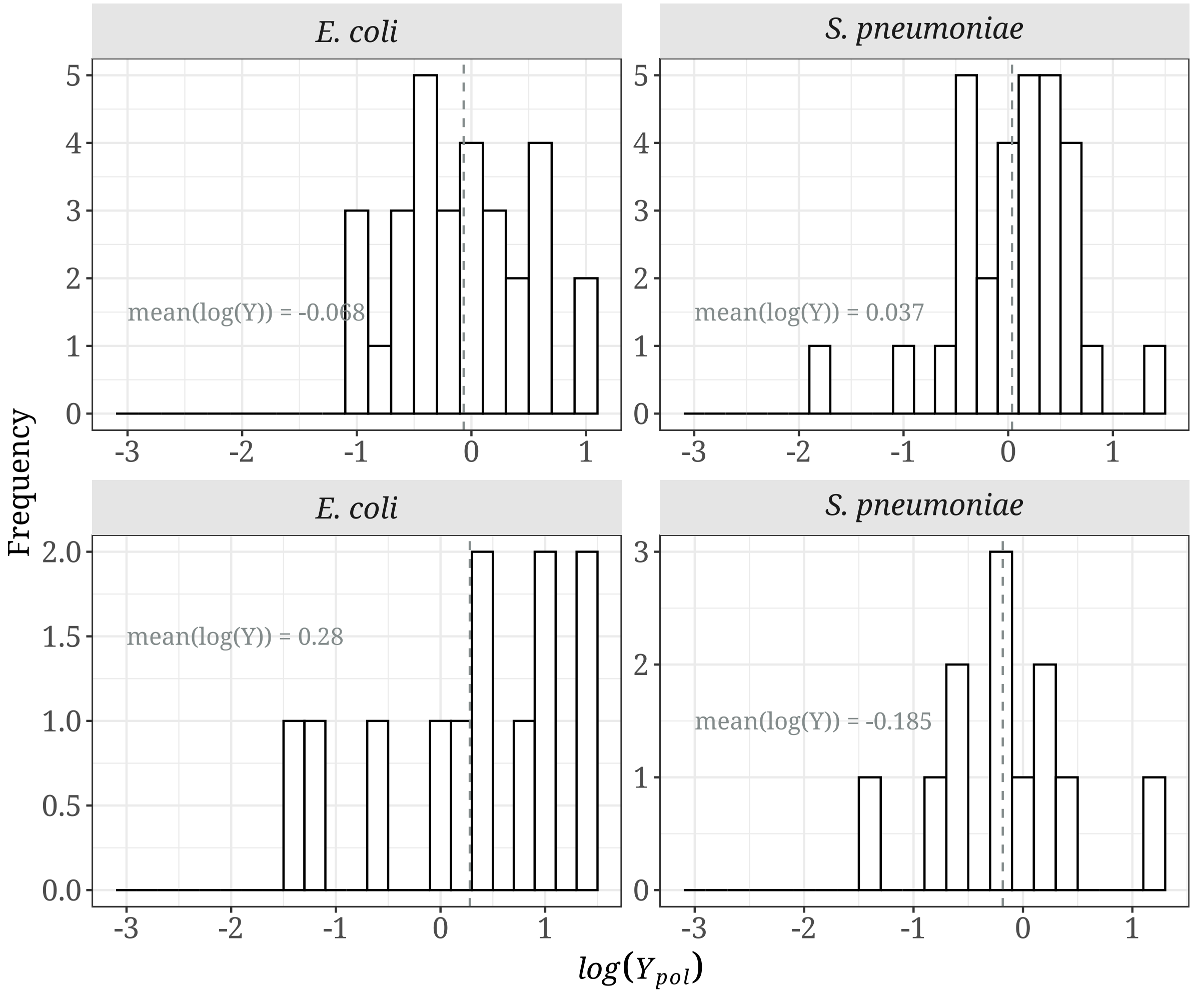

Figure S1. Distribution of *log(*Y_{pol}*)* in *E. coli* and *S. pneumoniae* for 2-fold (top) and 4-fold (bottom) amino acid combinations using more distantly related outgroups *E. fergusoni* and *S. mitis* to estimate the ancestral allele for *E. coli* and *S. pneumoniae*, respectively. The dashed line represents the mean value of the distribution. The binning size was set at 0.2. This analysis was performed with 30 and 12 amino acid mutations for 2-fold and 4-fold pairs, respectively, in each species.

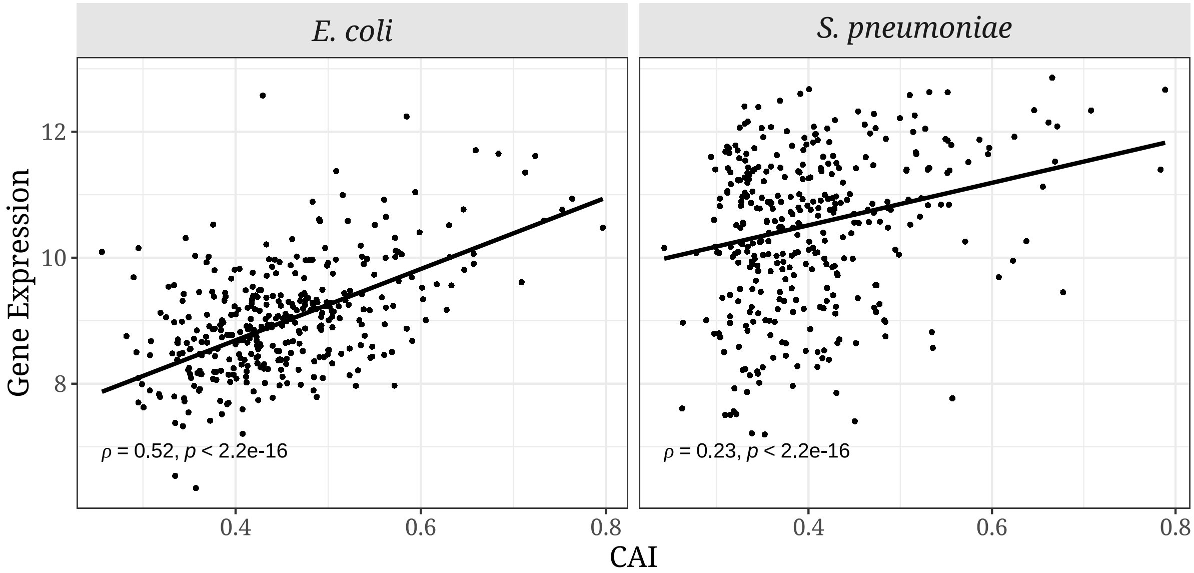

Figure S2. Relationship between gene expression and CAI per gene for *E. coli* and *S. pneumoniae*. The y-axis contains the mean gene expression values in each category (units in log RPKM). A linear model was fitted to the data and is represented with the dark line along with the Spearman's correlation coefficient and the respective significance values.
