# Supplementary material for: No Evidence that Selection on Synonymous Codon Usage Affects Patterns of Protein Evolution in Bacteria
Source: Genome Biol Evol. 2023 Dec 27;16(2):evad232. doi: 10.1093/gbe/evad232 (PMC10849182; doi:10.1093/gbe/evad232)
Supplement: evad232_Supplementary_Data [file evad232_supplementary_data.zip › FileS1.pdf]

Polymorphism analyses

afmoutinho

2023-12-02

This RMarkdown contains all analyses performed at the polymorphism level. The first chunk of the script reproduces the estimation of log(Y) in *E. coli* and *S. pneumoniae*.

```
#install.packages(c("coRdon", "dbplyr", "tidyverse", "plotly", "sysfonts"))

#if (!require("BiocManager", quietly = TRUE))
  #  install.packages("BiocManager")

#BiocManager::install("coRdon")

# Libraries
library(data.table)
library(dplyr)
library(plyr)
library(ggplot2)
library(ggpubr)
library(kableExtra)
library(tidyr)
library(coRdon)
library(tidyverse)
library(RGenetics)
library(cowplot)
library(Hmisc)
library(seqinr)
library(plotly)
library(stringi)
library(stringr)
##

## adding the font needed for the plot
library(showtext)
font_add_google("Noto Serif")
showtext_auto()
##

## need to add the rscu values from the ancestral allele
## table with all information per codon site:
codons_poly_all <- read.table(file = "~/Dropbox/CUB_supplementary_data/tables/polymorphims_tbl_all.csv"
                              sep = "\t", header = T)

# estimating the synonymous genetic divergence between E. coli and S. pneumoniae and the respective out
ds_df <- ddply(codons_poly_all, c("species", "outgroup"), function(x) {
```

```r
  ds <- sum(x$dS, na.rm = T)/sum(x$MeanNumberSynPosDiv, na.rm = T)
  data.frame(ds)
})
kable(ds_df)
```

species	outgroup	ds
ecoli	ealbertii	0.2620212
ecoli	efergusonii	0.2702313
spneumoniae	smitis	0.2159170
spneumoniae	spseudopneumoniae	0.1455534

```r
# keeping only the main outgroups
codons_poly_main <- subset(codons_poly_all, outgroup == "ealbertii" | outgroup == "spseudopneumoniae")

# this includes all alleles
write.table(codons_poly_main, file = "~/Dropbox/CUB_supplementary_data/tables/polymorphims_tbl_main.csv[illegible]
            sep = "\t", col.names = T, row.names = F, quote = F)
codons_poly_main <- read.table(file = "~/Dropbox/CUB_supplementary_data/tables/polymorphims_tbl_main.cs[illegible]
                               header = T)
## sites have filtered to keep only sites with more than 95% of seq data (156 out of 164 strains)

## counting numbers of monomorphic sites:
mono_sites <- as.data.frame(codons_poly_all[with(codons_poly_all,
                                                 NbAlleles == 1),])

mono_sites_counts <- ddply(mono_sites, c("species", "outgroup", "AncestralAllele"),
                           function(x) {
                             N <- nrow(x)
                             data.frame(N)
                           })

## counting the number of non-synonymous poly sites considering the aa change
# remove gene expression to account for those genes (missing data was making the results slightly diffe[illegible]
codons_poly_all2 <- subset(codons_poly_all, select = - mean_exp)
single_poly <- na.omit(codons_poly_all2[with(codons_poly_all2, NbAlleles == 2 & nt.dif == 1 &
                                     !(aa_ancestral == aa_derived)),])


## filter to keep only the mutations involving the same mutation
## for (i in 1:nrow(single_poly)) {
##   print(i)
##   aa1 <- strsplit(single_poly$AncestralAllele[i], split = "")
##   aa2 <- strsplit(single_poly$DerivedAllele[i], split = "")
##   comp.aa <- stri_compare(aa1[[1]], aa2[[1]])
##   j <- which(comp.aa != 0)
##   single_poly[i, "mutation"] <- paste0(aa1[[1]][j], aa2[[1]][j])
## }


## considering all mutations instead of only the ones that involve the ones with a change in RSCU
## limiting the decimal cases in RSCU values (it was not assuming it was the same value in some cases)
single_poly$log_rscu <- round(with(single_poly, log((rscu_derived + 1e-04)/(rscu_ancestral + 1e-04))),
                              digits = 4)
```

```
## counting the number of polymorphic sites for each codon mutation in each aa change:
poly_sites_counts <- ddply(single_poly, c("species", "outgroup", "aa_ancestral", "aa_derived",
                                          "aa_mut", "codon_mut", "AncestralAllele", "DerivedAllele",
                                          "rscu_ancestral", "rscu_derived",
                                          "log_rscu", "fold"), function(x) {
                             n_poly <- nrow(x)
                             data.frame(n_poly)
                           })


## combining the two tables:
df_counts_Y <- left_join(poly_sites_counts, mono_sites_counts,
                         by = c("species", "outgroup", "AncestralAllele"))

## removing aa mutations involving Met and Trp
sub_counts_Y <- subset(df_counts_Y, grepl("Met", aa_mut) == F &
                         grepl("Trp", aa_mut) == F)


# arranging the table to keep only what matters
counts_poly <- na.omit(subset(sub_counts_Y,
                              select = c("species", "outgroup", "aa_mut", "codon_mut", "aa_ancestral",
                                         "rscu_ancestral", "rscu_derived", "log_rscu",
                                         "n_poly", "N", "fold")))
counts_poly <- counts_poly[order(counts_poly$fold, decreasing = F),]

## checking how many aa mutations involve the same mutation type
#n_mut <- ddply(counts_poly, c("species", "outgroup", "aa_mut"), function(x) {
#  N <- length(unique(x$mutation))
#  data.frame(N)
#})
#n_mut1 <- n_mut[n_mut$N == 1,]

## subseting the table to keep only those with the same mutation type
#sub_counts_poly <- counts_poly[counts_poly$aa_mut %in% n_mut1$aa_mut,]

## only including the pairs of amino acids that involve the same mutation
write.csv(counts_poly, file = "~/Dropbox/CUB_supplementary_data/tables/TableS1.csv",
          col.names = T, row.names = F, quote = F)


## estimating logY based on the min and max change:
Y_df <- ddply(counts_poly, c("species", "outgroup", "aa_mut","aa_ancestral",
                              "aa_derived", "fold"),
              function(x) {
                # maximum increase in RSCU
                max_rscu <- which.max(x$log_rscu)
                # maximum decrease in RSCU
                min_rscu <- which.min(x$log_rscu)
                # log(Y)
                logY <- log((x$n_poly[max_rscu]*x$N[min_rscu])/(x$n_poly[min_rscu]*x$N[max_rscu]))
                # log(deltaRSCU)
                log_rscu <- round(log(((x$rscu_derived[max_rscu] + 1e-04)/(x$rscu_ancestral[max_rscu] +
```

```
                               ((x$rscu_derived[min_rscu] +  1e-04)/(x$rscu_ancestral[min_rscu] + 1e-04)
                data.frame(logY, log_rscu)
              })

## saving the table:
write.table(Y_df, file = "~/Dropbox/CUB_supplementary_data/tables/logY_codons_all.csv",
            sep = "\t", col.names = T, row.names = F, quote = F)

## for the main figures:
main_Y_df <- subset(Y_df, outgroup == "ealbertii" | outgroup == "spseudopneumoniae")
write.table(main_Y_df, file = "~/Dropbox/CUB_supplementary_data/tables/logY_codons_main.csv",
            sep = "\t", col.names = T, row.names = F, quote = F)
```

Distribution of log(Y) across codons in each species with the main outgroups (E. albertii and S. pseudopneumoniae).

```
main_Y_df <- read.table(file = "~/Dropbox/CUB_supplementary_data/tables/logY_codons_main.csv",
                        sep = "\t", header = T)

# keeping only 2-fold and 4-fold sites
main_Y_df2 <- main_Y_df[is.na(main_Y_df$fold) == F,]
main_Y_df2$species <- factor(main_Y_df2$species, levels = c("ecoli", "spneumoniae"))
levels(main_Y_df2$species) <- c(expression(italic("E. coli")),
                               expression(italic("S. pneumoniae")))

# distribution of log(Y)
plot_distY <- ggplot(main_Y_df2, aes(x=logY)) +
  geom_histogram(aes(y=..count..),
                 binwidth=.2,
                 colour="black", fill="white") +
  xlab(expression(atop(italic(log(Y[pol]))))) +
  ylab("Frequency") +
  geom_vline(data = ddply(main_Y_df2, c("species", "fold"), summarise, avg = mean(logY)),
             aes(xintercept=avg),linetype="dashed",
             color = "azure4", size=0.5) +
  facet_wrap(fold~species, scales = "free", labeller = function(x) {label_parsed(x[2])}) +
  geom_text(data = ddply(main_Y_df2, c("species", "fold"), summarise, avg = mean(logY)),
            aes(x = -3, y = 2, label = paste("mean(log(Y)) = ",
                                             round(avg, digits = 3), sep = "")),
            hjust = 0,family = "Noto Serif", size = 4, color = "azure4") +
  theme_bw() +
  theme(text = element_text(family = "Noto Serif", size = 14),
        axis.text = element_text(family = "Noto Serif", size = 14),
        strip.text.x = element_text(family = "Noto Serif", face = "bold", size = 14),
        strip.background.x = element_rect(fill = "gray90", linetype = "blank"))
plot_distY
```

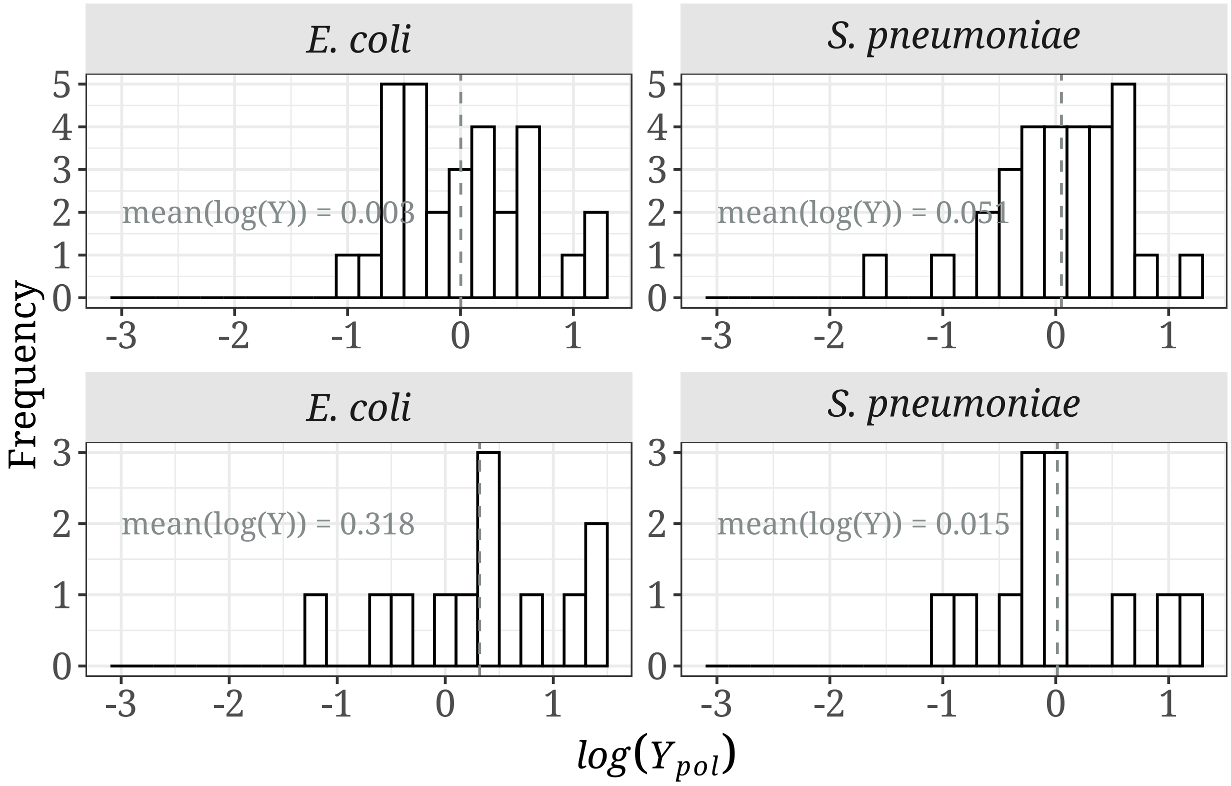


```
#dir.create("~/path-to/CUB_supplementary_data/Figures/")
ggsave(filename = "Figure1.pdf", plot = plot_distY, device = "pdf",
       path = "~/Dropbox/CUB_supplementary_data/Figures/", width = 8, height = 7, units = "in")


# checking if the mean is different form 0:
p_logY_folds <- ddply(main_Y_df2, c("species", "fold"), function(x) {
  t_test <- t.test(x$logY)
  mean <- t_test$estimate
  p_value <- t_test$p.value
  data.frame(mean, p_value)
})
kable(p_logY_folds)
```

species	fold	mean	p_value
italic("E. coli")	2-fold	0.0026423	0.9806270
italic("E. coli")	4-fold	0.3182192	0.1993857
italic("S. pneumoniae")	2-fold	0.0507607	0.6406617
italic("S. pneumoniae")	4-fold	0.0146976	0.9375055

```
# not significant
```

Distribution of log(Y) across codons in each species with the other outgroups (E. fergusoni and S. mitis).

```r
Y_df <- read.table(file = "~/Dropbox/CUB_supplementary_data/tables/logY_codons_all.csv",
                        sep = "\t", header = T)

other_outs <- subset(Y_df, outgroup == "efergusonii" | outgroup == "smitis")

# keeping only 2-fold and 4-fold sites
other_outs2 <- other_outs[is.na(other_outs$fold) == F,]
other_outs2$species <- factor(other_outs2$species, levels = c("ecoli", "spneumoniae"))
levels(other_outs2$species) <- c(expression(italic("E. coli")),
                               expression(italic("S. pneumoniae")))

# distribution of log(Y)
plot_distY_other <- ggplot(other_outs2, aes(x=logY)) +
  geom_histogram(aes(y=..count..),
                 binwidth=.2,
                 colour="black", fill="white") +
  xlab(expression(atop(italic(log(Y[pol]))))) +
  ylab("Frequency") +
  geom_vline(data = ddply(other_outs2, c("species", "fold"), summarise, avg = mean(logY)),
             aes(xintercept=avg),linetype="dashed",
             color = "azure4", size=0.5) +
  facet_wrap(fold~species, scales = "free", labeller = function(x) {label_parsed(x[2])}) +
  geom_text(data = ddply(other_outs2, c("species", "fold"), summarise, avg = mean(logY)),
            aes(x = -3, y = 1.5, label = paste("mean(log(Y)) = ",
                                               round(avg, digits = 3), sep = "")),
            hjust = 0,family = "Noto Serif", size = 4, color = "azure4") +
  theme_bw() +
  theme(text = element_text(family = "Noto Serif", size = 14),
        axis.text = element_text(family = "Noto Serif", size = 14),
        strip.text.x = element_text(family = "Noto Serif", face = "bold", size = 14),
        strip.background.x = element_rect(fill = "gray90", linetype = "blank"))
plot_distY_other
```

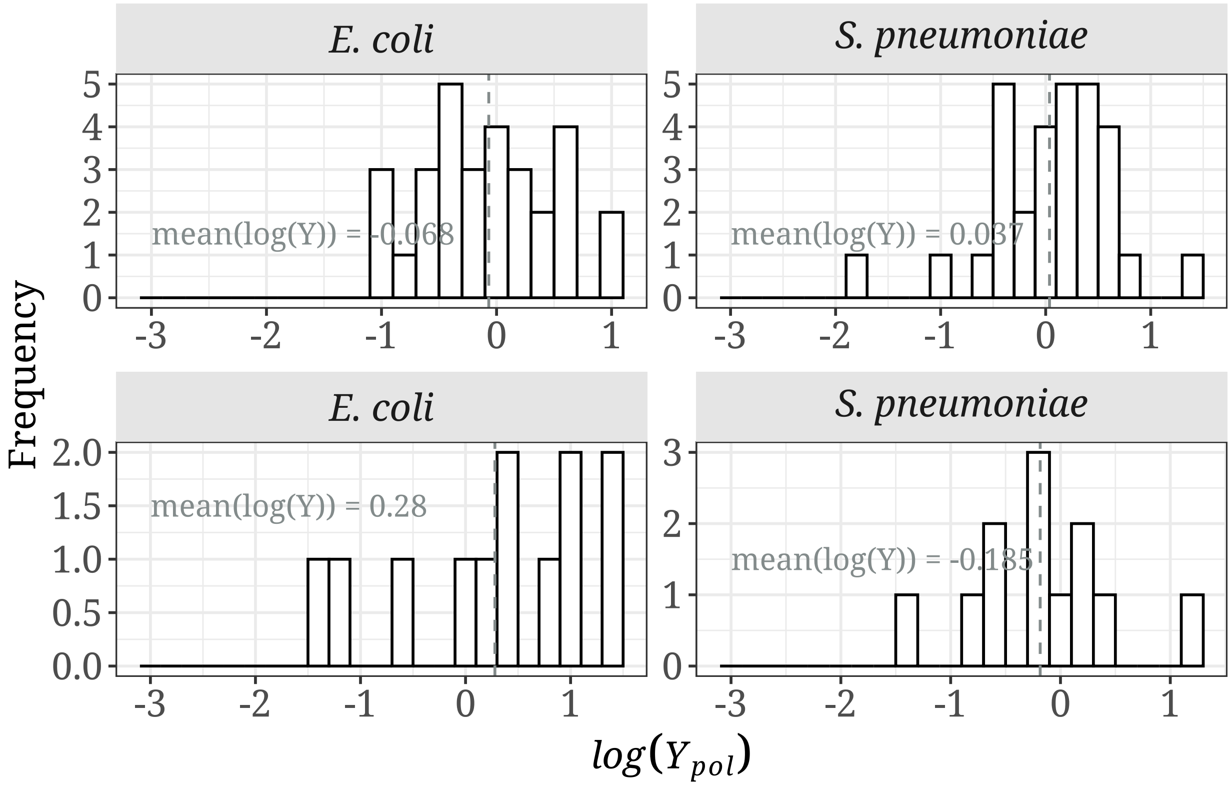


```
#dir.create("~/Dropbox/CUB_supplementary_data/Figures/Revision3/")
ggsave(filename = "FigureS1.pdf", plot = plot_distY_other, device = "pdf",
       path = "~/Dropbox/CUB_supplementary_data/Figures/", width = 8, height = 7, units = "in")


# checking if the mean is different form 0:
p_logY_folds_other <- ddply(other_outs2, c("species", "fold"), function(x) {
  t_test <- t.test(x$logY)
  mean <- t_test$estimate
  p_value <- t_test$p.value
  data.frame(mean, p_value)
})
kable(p_logY_folds_other)
```

species	fold	mean	p_value
italic("E. coli")	2-fold	-0.0675924	0.5247071
italic("E. coli")	4-fold	0.2795113	0.3150825
italic("S. pneumoniae")	2-fold	0.0366213	0.7470147
italic("S. pneumoniae")	4-fold	-0.1846919	0.3477540

Relationship between log(Y) and log(var_rscu)

```
### log(Y) ~ log(rscu_p/rscu_u)
p.RSCU.logY <- ggplot(main_Y_df2, aes(log_rscu, logY, label = aa_mut)) +
    geom_point(size = 0.6) +
    geom_smooth(method = "glm", formula = y~x, se = T, color = "black", size = 1) +
```

```
    xlab(expression(paste("log(", italic(Delta*RSCU), ")", sep = ""))) +
    ylab(expression(atop(italic(log(Y[pol]))))) +
    stat_cor(label.x.npc = "left", label.y.npc = "bottom", method = "spearman", size = 3.5,
             cor.coef.name = "rho") +
    facet_wrap(fold~species, scales = "free", labeller = function(x) {label_parsed(x[2])}) +
    #facet_grid(fold~species, scales = "free", labeller = label_parsed) +
    theme_bw() +
    theme(text = element_text(family = "Noto Serif", size = 14),
          strip.text.x = element_text(family = "Noto Serif", size = 14),
          strip.text.y = element_text(family = "Noto Serif", size = 14),
          strip.background = element_rect(fill = "gray90", linetype = "blank"))
p.RSCU.logY
```

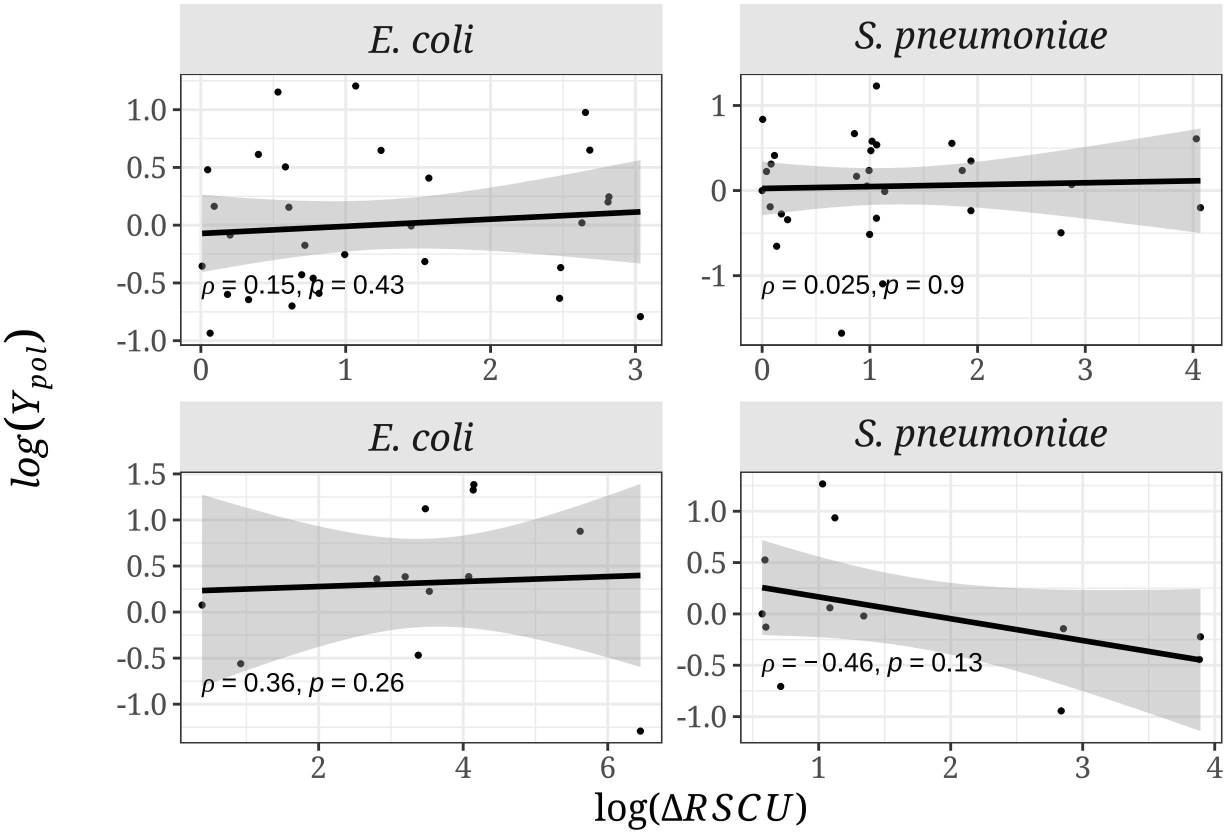


```
ggsave(filename = "Figure2.pdf", plot = p.RSCU.logY, device = "pdf",
       path = "~/Dropbox/CUB_supplementary_data/Figures/", width = 8.13, height = 6.42, units = "in")
```

log(Y) analysis taking into account codon adaptation index (CAI)

```
# assessing the relationship between CAI and gene expression
cai_exp_df <- na.omit(subset(codons_poly_main, select = c("species", "cai_major", "mean_exp")))

# plotting the relationship:
cai_exp_df$species <- factor(cai_exp_df$species, levels = c("ecoli", "spneumoniae"))
levels(cai_exp_df$species) <- c(expression(italic("E. coli")),
                                expression(italic("S. pneumoniae")))
```

```r
p.cai.exp <- ggplot(cai_exp_df, aes(cai_major, mean_exp)) +
    geom_point(size = 0.6) +
    geom_smooth(method = "glm", formula = y~x, se = T, color = "black", size = 1) +
    xlab("CAI") +
    ylab("Gene Expression") +
    stat_cor(label.x.npc = "left", label.y.npc = "bottom", method = "spearman", size = 3.5,
             cor.coef.name = "rho") +
    facet_grid(~species, scales = "free", labeller = label_parsed) +
    #facet_grid(fold~species, scales = "free", labeller = label_parsed) +
    theme_bw() +
    theme(text = element_text(family = "Noto Serif", size = 14),
          strip.text.x = element_text(family = "Noto Serif", size = 14),
          strip.text.y = element_text(family = "Noto Serif", size = 14),
          strip.background = element_rect(fill = "gray90", linetype = "blank"))
p.cai.exp
```

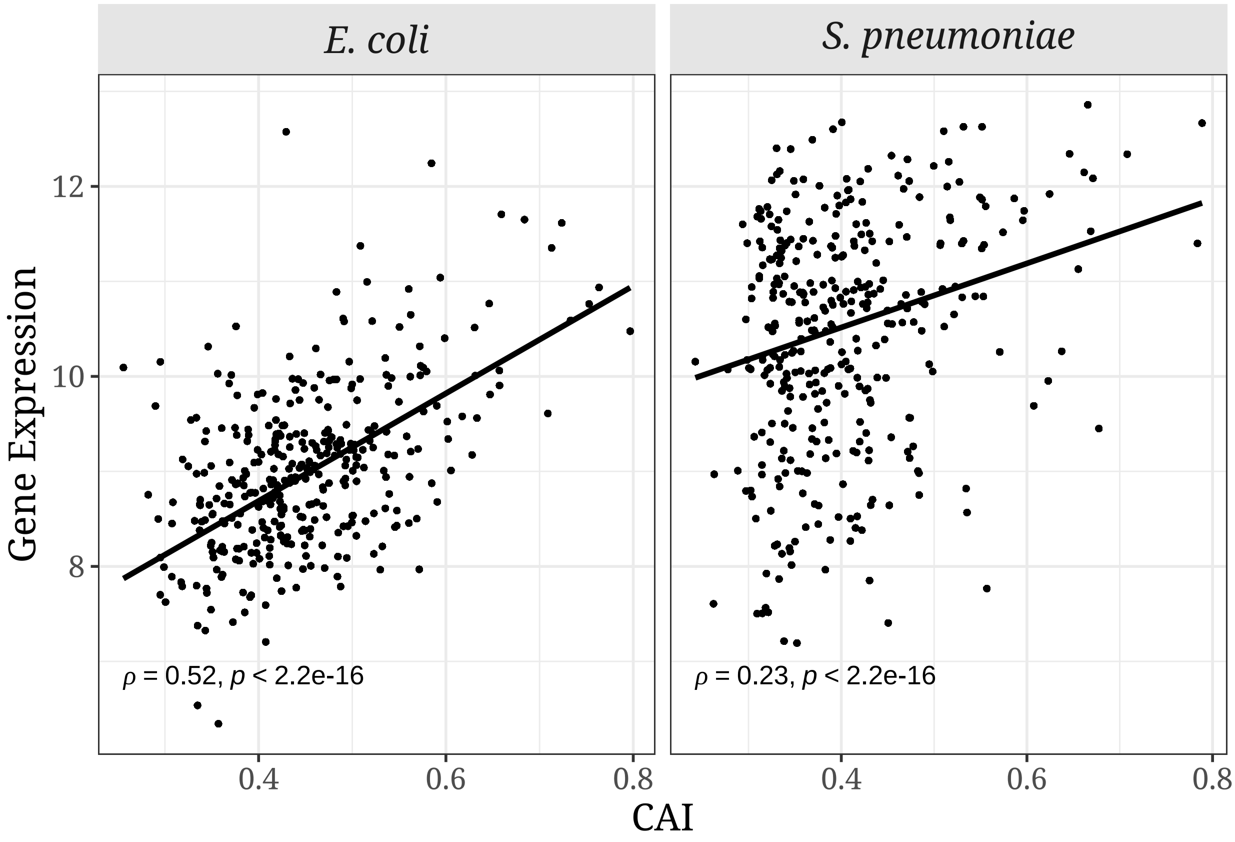


```r
ggsave(filename = "FigureS2.pdf", plot = p.cai.exp, device = "pdf",
       path = "~/Dropbox/CUB_supplementary_data/Figures/", width = 8.13, height = 4, units = "in")


# removing the sites without expression information (NA values)
codons_poly_main <- ddply(codons_poly_main, "species", function(x) {
  x$cai_cat <- as.numeric(cut2(x$cai_major, g = 4))
  return(x)
})
```

```r
# removing missing data:
cai_df <- subset(codons_poly_main, is.na(codons_poly_main$cai_cat) == F)

# split data.frame into each expression category in each species
splits_df <- cai_df %>%
  split(list(cai_df$species, cai_df$cai_cat), drop = T)

# get all codons per expression category (taking the major allele)
codon_cai_ingroup <- lapply(splits_df, function(x) {
  unlist(str_split(x$DerivedAllele, ""))
})


# get RSCU for each cai category:
uco_cai_ingroup <- list()
for (i in 1:length(codon_cai_ingroup)) {
  uco_cai_ingroup[[i]] <- uco(codon_cai_ingroup[[i]], as.data.frame = TRUE)
  uco_cai_ingroup[[i]]$species <- str_split_fixed(names(codon_cai_ingroup[i]), "\\.", 2)[,1]
  uco_cai_ingroup[[i]]$cai_cat <- as.numeric(str_split_fixed(names(codon_cai_ingroup[i]), "\\.", 2)[,2]
}

# combining all tables:
df_uco_cai_ingroup <- rbindlist(uco_cai_ingroup)

# keeping only columns of interest:
df_uco_cai_ingroup <- subset(df_uco_cai_ingroup, select = - c(eff, freq))
df_uco_cai_ingroup$codon <- toupper(df_uco_cai_ingroup$codon)

## doing the same for the ancestral allele:
codon_cai_ancestral <- lapply(splits_df, function(x) {
  unlist(str_split(x$AncestralAllele, ""))
})

uco_cai_ancestral <- list()
for (i in 1:length(codon_cai_ancestral)) {
  uco_cai_ancestral[[i]] <- uco(codon_cai_ancestral[[i]], as.data.frame = TRUE)
  uco_cai_ancestral[[i]]$species <- str_split_fixed(names(codon_cai_ancestral[i]), "\\.", 2)[,1]
  uco_cai_ancestral[[i]]$cai_cat <- as.numeric(str_split_fixed(names(codon_cai_ancestral[i]), "\\.", 2)
}

# combining all tables:
df_uco_cai_ancestral <- rbindlist(uco_cai_ancestral)

# keeping only columns of interest:
df_uco_cai_ancestral <- subset(df_uco_cai_ancestral, select = - c(eff, freq))
df_uco_cai_ancestral$codon <- toupper(df_uco_cai_ancestral$codon)

### merging this table with the poly codons:

# subsetting the main data set to include only columns of interest:
poly_codons_cai <- subset(codons_poly_main, select = c("species", "coID", "aa_ancestral",
                                                       "aa_derived", "AncestralAllele", "DerivedAllele"
```

```
                                                       "NbAlleles", "nt.dif", "aa_mut", "fold",
                                                       "codon_mut", "cai_major", "cai_cat"))

# merging with major allele:
colnames(df_uco_cai_ancestral)[c(1,2,3)] <- c("aa_ancestral", "AncestralAllele", "rscu_ancestral")
poly_codons_cai <- left_join(poly_codons_cai, df_uco_cai_ancestral, by = c("species", "cai_cat",
                                                                  "aa_ancestral", "AncestralAllele"))
# merging with minor allele:
colnames(df_uco_cai_ingroup)[c(1,2,3)] <- c("aa_derived", "DerivedAllele", "rscu_derived")
poly_codons_cai <- left_join(poly_codons_cai, df_uco_cai_ingroup, by = c("species", "cai_cat",
                                                                  "aa_derived", "DerivedAllele"))
poly_codons_cai <- na.omit(poly_codons_cai)


# estimating the change in RSCU values in each expression category
# adding 1e-04 for cases where RSCU = 0
poly_codons_cai$log_rscu <- with(poly_codons_cai, log((rscu_derived + 1e-04)/(rscu_ancestral + 1e-04)))


# counts of monomorphic sites:
mono_sites_cai <- poly_codons_cai[with(poly_codons_cai, NbAlleles == 1),]

mono_sites_cai_counts <- ddply(mono_sites_cai, c("species", "cai_cat", "AncestralAllele"),
                               function(x) {
                                 N <- nrow(x)
                                 data.frame(N)
                                 })

# poly counts
# counting the number of non-synonymous poly sites considering the aa transition
single_poly_cai <- poly_codons_cai[with(poly_codons_cai, NbAlleles == 2 & nt.dif == 1
                                        & !(aa_ancestral == aa_derived)),]

## filter to keep only the mutations involving the same mutation *HERE*
#for (i in 1:nrow(single_poly_cai)) {
#  print(i)
#  aa1 <- strsplit(single_poly_cai$AncestralAllele[i], split = "")
#  aa2 <- strsplit(single_poly_cai$DerivedAllele[i], split = "")
#  comp.aa <- stri_compare(aa1[[1]], aa2[[1]])
#  j <- which(comp.aa != 0)
#  single_poly_cai[i, "mutation"] <- paste0(aa1[[1]][j], aa2[[1]][j])
#}


# getting gene expression data to estimate mean expression in each category
cai_df <- subset(single_poly_cai, select = c("cai_major", "cai_cat", "species"))
cai_df2 <- ddply(cai_df, c("species", "cai_cat"), function(x) {
  mean_cai <- mean(x$cai_major)
  data.frame(mean_cai)
})
colnames(cai_df2)[2] <- "category"

# doing the counts for each expression category:
```

```
poly_sites_cai_counts <- ddply(single_poly_cai, c("species", "cai_cat", "fold",
                                                  "aa_ancestral", "aa_derived", "AncestralAllele",
                                                  "DerivedAllele", "aa_mut", "codon_mut", "rscu_ancestra
                                                  "rscu_derived", "log_rscu"),
                               function(x) {
                                 n_poly <- nrow(x)
                                 data.frame(n_poly)
                                 })

# combining the two tables:
df_counts_Y_cai <- left_join(poly_sites_cai_counts, mono_sites_cai_counts,
                             by = c("species", "AncestralAllele", "cai_cat"))

# removing Met and Trp codons:
df2_counts_Y_cai <- subset(df_counts_Y_cai, grepl("Met", aa_mut) == F &
                             grepl("Trp", aa_mut) == F)


df2_counts_Y_cai <- df2_counts_Y_cai[order(df2_counts_Y_cai$fold, df2_counts_Y_cai$species,
                                           df2_counts_Y_cai$cai_cat, decreasing = F),]
df2_counts_Y_cai$variable <- rep("CAI", nrow(df2_counts_Y_cai))
colnames(df2_counts_Y_cai)[2] <- "category"

## checking how many aa mutations involve the same mutation type
#n_mut <- ddply(df2_counts_Y_cai, c("species", "aa_mut"), function(x) {
#  N <- length(unique(x$mutation))
#  data.frame(N)
#})
#n_mut1 <- n_mut[n_mut$N == 1,]

## subseting the table to keep only those with the same mutation type
#sub_counts_Y_cai <- df2_counts_Y_cai[df2_counts_Y_cai$aa_mut %in% n_mut1$aa_mut,]

write.table(df2_counts_Y_cai, file = "~/Dropbox/CUB_supplementary_data/tables/TableS4.csv",
            sep = "\t", col.names = T,row.names = F)


# estimating Y using the max/min approach:
Y_df_cai <- ddply(df2_counts_Y_cai, c("species", "category", "aa_mut",
                                      "aa_ancestral", "aa_derived", "fold"),
                  function(x) {
                    # maximum increase in RSCU
                    max_rscu <- which.max(x$log_rscu)
                    # maximum decrease in RSCU
                    min_rscu <- which.min(x$log_rscu)
                    # keep only amino acid mutations that have at least 2 codon mutations
                    if (max_rscu != min_rscu) {
                      logY <- log((x$n_poly[max_rscu]*x$N[min_rscu])/(x$n_poly[min_rscu]*x$N[max_r
                      log_rscu <- round(log(((x$rscu_derived[max_rscu] + 1e-04)/(x$rscu_ancestral[
                          ((x$rscu_derived[min_rscu] +  1e-04)/(x$rscu_ancestral[min_rscu] + 1e-04
                    } else {
                      logY <- NA
                      log_rscu <- NA
```

```r
                          }
                          data.frame(logY, log_rscu)
                        }
)

# combining with the mean expression data:
Y_df_cai2 <- left_join(Y_df_cai, cai_df2, by = c("species", "category"))

# saving the table: -- add this info in Table SX
write.table(Y_df_cai, file = "~/Dropbox/CUB_supplementary_data/tables/logY_cai.csv",
            sep = "\t", col.names = T, row.names = F, quote = F)
```

```r
#Y_df_cai <- read.table(file = "~/Dropbox/CUB_supplementary_data/tables/logY_cai.csv",
#                             sep = "\t", header = T)

## keeping only 2-folds and 4-folds
Y_df_cai2 <- na.omit(Y_df_cai)

# logY ~ log(rscu) + CAI
Y_df_cai2$species <- factor(Y_df_cai2$species, levels = c("ecoli", "spneumoniae"))
levels(Y_df_cai2$species) <- c(expression(italic("E. coli")),
                               expression(italic("S. pneumoniae")))

# checking the relationship between log(RSCU_max/RSCU_min) and log(Y)
p.RSCU.logY_cai <- ggplot(Y_df_cai2, aes(log_rscu, logY, label = aa_mut,
                                          col=as.ordered(category))) +
  geom_point(size = 0.6) +
  geom_smooth(method = "glm", formula = y~x, se = F, size = 1) +
  xlab(expression(paste("log(", italic(Delta*RSCU), ")", sep = ""))) +
  ylab(expression(atop(italic(log(Y[pol]))))) +
  guides(col=guide_legend(title="CAI")) +
  #facet_grid(fold~species, scales = "free", labeller = label_parsed) +
  facet_wrap(fold~species, scales = "free", labeller = function(x) {label_parsed(x[2])}) +
  theme_bw() +
  theme(text = element_text(family = "Noto Serif", size = 12),
        strip.text.x = element_text(family = "Noto Serif", size = 12),
        strip.text.y = element_text(family = "Noto Serif", face = "bold", size = 12),
        strip.background.x = element_rect(fill = "gray90", linetype = "blank"),
        strip.background.y = element_rect(fill = "gray90", linetype = "blank"))
p.RSCU.logY_cai
```

```
## Warning: The following aesthetics were dropped during statistical transformation: label
## i This can happen when ggplot fails to infer the correct grouping structure in
##   the data.
## i Did you forget to specify a `group` aesthetic or to convert a numerical
##   variable into a factor?
## The following aesthetics were dropped during statistical transformation: label
## i This can happen when ggplot fails to infer the correct grouping structure in
##   the data.
## i Did you forget to specify a `group` aesthetic or to convert a numerical
##   variable into a factor?
## The following aesthetics were dropped during statistical transformation: label
## i This can happen when ggplot fails to infer the correct grouping structure in
```

```
##   the data.
## i Did you forget to specify a ‘group‘ aesthetic or to convert a numerical
##   variable into a factor?
## The following aesthetics were dropped during statistical transformation: label
## i This can happen when ggplot fails to infer the correct grouping structure in
##   the data.
## i Did you forget to specify a ‘group‘ aesthetic or to convert a numerical
##   variable into a factor?
```

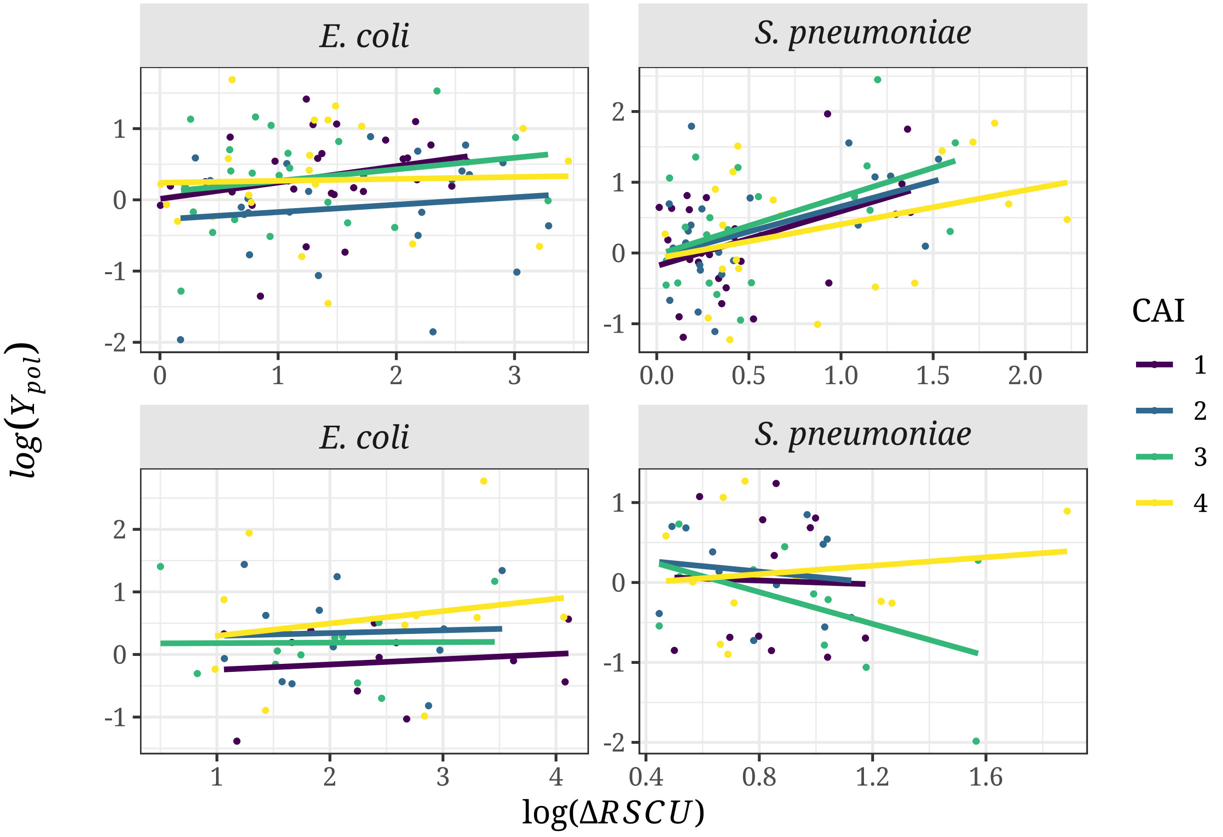


```r
ggsave(filename = "Figure5.pdf", plot = p.RSCU.logY_cai, device = "pdf",
       path = "~/Dropbox/CUB_supplementary_data/Figures/", width = 8.84,
       height = 6.68, units = "in")
```

```
## Warning: The following aesthetics were dropped during statistical transformation: label
## i This can happen when ggplot fails to infer the correct grouping structure in
##   the data.
## i Did you forget to specify a ‘group‘ aesthetic or to convert a numerical
##   variable into a factor?
## The following aesthetics were dropped during statistical transformation: label
## i This can happen when ggplot fails to infer the correct grouping structure in
##   the data.
## i Did you forget to specify a ‘group‘ aesthetic or to convert a numerical
##   variable into a factor?
## The following aesthetics were dropped during statistical transformation: label
## i This can happen when ggplot fails to infer the correct grouping structure in
```

```
##   the data.
## i Did you forget to specify a `group` aesthetic or to convert a numerical
##   variable into a factor?
## The following aesthetics were dropped during statistical transformation: label
## i This can happen when ggplot fails to infer the correct grouping structure in
##   the data.
## i Did you forget to specify a `group` aesthetic or to convert a numerical
##   variable into a factor?
```

```
## linear regressions:
stats_cai <- ddply(Y_df_cai2, c("species", "fold", "category"), function(x) {
  cor_test <- cor.test(x$logY, x$log_rscu, method = "spearman", exact = FALSE)
  rho <- cor_test$estimate
  p_value <- cor_test$p.value
  N <- nrow(x)
  data.frame(rho, p_value, N)
})
kable(stats_cai)
```

species	fold	category	rho	p_value	N
italic("E. coli")	2-fold	1	0.3089050	0.1029979	29
italic("E. coli")	2-fold	2	0.1307692	0.5332475	25
italic("E. coli")	2-fold	3	0.2165217	0.3095199	24
italic("E. coli")	2-fold	4	0.1208185	0.6018966	21
italic("E. coli")	4-fold	1	0.0209790	0.9484022	12
italic("E. coli")	4-fold	2	0.0769231	0.8121827	12
italic("E. coli")	4-fold	3	-0.0272727	0.9365584	11
italic("E. coli")	4-fold	4	0.1636364	0.6306852	11
italic("S. pneumoniae")	2-fold	1	0.1405128	0.4935653	26
italic("S. pneumoniae")	2-fold	2	0.3063241	0.1551435	23
italic("S. pneumoniae")	2-fold	3	0.4150198	0.0547773	22
italic("S. pneumoniae")	2-fold	4	0.3308271	0.1542360	20
italic("S. pneumoniae")	4-fold	1	-0.0699301	0.8290236	12
italic("S. pneumoniae")	4-fold	2	-0.2027972	0.5273024	12
italic("S. pneumoniae")	4-fold	3	-0.3909091	0.2345401	11
italic("S. pneumoniae")	4-fold	4	0.0545455	0.8734466	11

```
# nothing is significant
```

Linear models:

```
## doing the linear model accounting for standard error:
## will do the linear models only for 2-folds (4-folds have too little data points)
ecoli_logY_cai_2fold <- subset(Y_df_cai2, species == 'italic("E. coli")' & fold == "2-fold")
ecoli_logY_cai_4fold <- subset(Y_df_cai2, species == 'italic("E. coli")' & fold == "4-fold")
spneu_logY_cai_2fold <- subset(Y_df_cai2, species == 'italic("S. pneumoniae")' & fold == "2-fold")
spneu_logY_cai_4fold <- subset(Y_df_cai2, species == 'italic("S. pneumoniae")' & fold == "4-fold")

## E. coli
## 2-folds
lm_ecoli_2fold <- with(ecoli_logY_cai_2fold, lm(logY ~ log_rscu + category))
lm_ecoli2_2fold <- with(ecoli_logY_cai_2fold, lm(logY ~ log_rscu * category))
AIC(lm_ecoli_2fold, lm_ecoli2_2fold) # best without interaction
```

```
##                 df      AIC
## lm_ecoli_2fold   4 217.5823
## lm_ecoli2_2fold  5 219.2858
```

```r
summary(lm_ecoli_2fold)
```

```
##
## Call:
## lm(formula = logY ~ log_rscu + category)
##
## Residuals:
##     Min      1Q  Median      3Q     Max
## -2.1185 -0.3425  0.0876  0.4484  1.5224
##
## Coefficients:
##             Estimate Std. Error t value Pr(>|t|)
## (Intercept)  0.04784    0.21010   0.228    0.820
## log_rscu     0.07922    0.08244   0.961    0.339
## category     0.01720    0.06412   0.268    0.789
##
## Residual standard error: 0.7082 on 96 degrees of freedom
## Multiple R-squared:  0.009839,   Adjusted R-squared:  -0.01079
## F-statistic: 0.477 on 2 and 96 DF,  p-value: 0.6221
```

```r
# information in TableS1
#            Estimate Std. Error t value Pr(>|t|)
#(Intercept)  0.04784    0.21010   0.228    0.820
#log_rscu     0.07922    0.08244   0.961    0.339
#cai_cat      0.01720    0.06412   0.268    0.789

## 4-folds
lm_ecoli_4fold <- with(ecoli_logY_cai_4fold, lm(logY ~ log_rscu + category))
lm_ecoli2_4fold <- with(ecoli_logY_cai_4fold, lm(logY ~ log_rscu * category))
AIC(lm_ecoli_4fold, lm_ecoli2_4fold) # best without interaction
```

```
##                 df      AIC
## lm_ecoli_4fold   4 114.1866
## lm_ecoli2_4fold  5 115.9679
```

```r
summary(lm_ecoli_4fold)
```

```
##
## Call:
## lm(formula = logY ~ log_rscu + category)
##
## Residuals:
##      Min       1Q   Median       3Q      Max
## -1.58156 -0.52270 -0.05512  0.45907  2.12309
##
## Coefficients:
##             Estimate Std. Error t value Pr(>|t|)
## (Intercept)  -0.4491     0.4182  -1.074   0.2889
```

```
## log_rscu      0.0945     0.1283   0.737   0.4654
## category      0.1948     0.1052   1.851   0.0711 .
## ---
## Signif. codes:  0 '***' 0.001 '**' 0.01 '*' 0.05 '.' 0.1 ' ' 1
##
## Residual standard error: 0.7937 on 43 degrees of freedom
## Multiple R-squared:  0.07999,    Adjusted R-squared:  0.0372
## F-statistic: 1.869 on 2 and 43 DF,  p-value: 0.1665
```

```r
#             Estimate Std. Error t value Pr(>|t|)
#(Intercept)   -0.4491     0.4182  -1.074   0.2889
#log_rscu      0.0945     0.1283   0.737   0.4654
#cai_cat       0.1948     0.1052   1.851   0.0711 .


## S. pneumoniae
## 2-folds
lm_spneu_2fold <- with(spneu_logY_cai_2fold, lm(logY ~ log_rscu + category))
lm_spneu2_2fold <- with(spneu_logY_cai_2fold, lm(logY ~ log_rscu * category))
AIC(lm_spneu_2fold, lm_spneu2_2fold) # best without interaction
```

```
##                 df      AIC
## lm_spneu_2fold   4 206.6637
## lm_spneu2_2fold  5 207.6836
```

```r
summary(lm_spneu_2fold)
```

```
##
## Call:
## lm(formula = logY ~ log_rscu + category)
##
## Residuals:
##      Min       1Q   Median       3Q      Max
## -1.48131 -0.45352  0.00909  0.52319  1.75884
##
## Coefficients:
##             Estimate Std. Error t value Pr(>|t|)
## (Intercept) -0.03947    0.18704  -0.211    0.833
## log_rscu     0.63767    0.14996   4.252 5.26e-05 ***
## category    -0.01120    0.07116  -0.157    0.875
## ---
## Signif. codes:  0 '***' 0.001 '**' 0.01 '*' 0.05 '.' 0.1 ' ' 1
##
## Residual standard error: 0.733 on 88 degrees of freedom
## Multiple R-squared:  0.1779, Adjusted R-squared:  0.1592
## F-statistic: 9.519 on 2 and 88 DF,  p-value: 0.0001809
```

```r
# information in TableS1
#             Estimate Std. Error t value Pr(>|t|)
#(Intercept) -0.03947    0.18704  -0.211    0.833
#log_rscu     0.63767    0.14996   4.252 5.26e-05 ***
#cai_cat     -0.01120    0.07116  -0.157    0.875
```

```r
## 4-folds
lm_spneu_4fold <- with(spneu_logY_cai_4fold, lm(logY ~ log_rscu + category))
lm_spneu2_4fold <- with(spneu_logY_cai_4fold, lm(logY ~ log_rscu * category))
AIC(lm_spneu_4fold, lm_spneu2_4fold) # best without interaction
```

```
##                  df      AIC
## lm_spneu_4fold   4 107.7466
## lm_spneu2_4fold  5 109.2488
```

```r
summary(lm_spneu_4fold)
```

```
##
## Call:
## lm(formula = logY ~ log_rscu + category)
##
## Residuals:
##      Min       1Q   Median       3Q      Max
## -1.73172 -0.67342 -0.05157  0.55391  1.26452
##
## Coefficients:
##              Estimate Std. Error t value Pr(>|t|)
## (Intercept)  0.327752   0.386621   0.848    0.401
## log_rscu    -0.374431   0.354620  -1.056    0.297
## category     0.001829   0.098262   0.019    0.985
##
## Residual standard error: 0.7401 on 43 degrees of freedom
## Multiple R-squared:  0.02548,    Adjusted R-squared:  -0.01984
## F-statistic: 0.5622 on 2 and 43 DF,  p-value: 0.5741
```

```r
#             Estimate Std. Error t value Pr(>|t|)
#(Intercept)  0.327752   0.386621   0.848    0.401
#log_rscu    -0.374431   0.354620  -1.056    0.297
#cai_cat      0.001829   0.098262   0.019    0.985

### estimating the slopes of the correlations:
slope_cai <- ddply(Y_df_cai2, c("species", "fold", "category"), function(x) {
  lm_cai <- lm(x$logY ~ x$log_rscu)
  slope <- coef(lm_cai)[2]
  data.frame(slope)
})
# generally lower slopes for higher CAI categories
```
